# Epiphytic PGPB *Bacillus megaterium* AFI1 and *Paenibacillus nicotianae* AFI2 Improve Wheat Growth and Antioxidant Status under Ni Stress

**DOI:** 10.3390/plants10112334

**Published:** 2021-10-29

**Authors:** Veronika N. Pishchik, Polina S. Filippova, Galina V. Mirskaya, Yuriy V. Khomyakov, Vitaliy E. Vertebny, Viktoriya I. Dubovitskaya, Yuliya V. Ostankova, Aleksandr V. Semenov, Debasis Chakrabarty, Evgeny V. Zuev, Vladimir K. Chebotar

**Affiliations:** 1 All-Russia Research Institute for Agricultural Microbiology, Podbelskogo hwy, 3, Pushkin, 196608 St. Petersburg, Russia; 2 Agrophysical Scientific Research Institute, Grazhdansky pr. 14, 195220 St. Petersburg, Russia; galinanm@gmail.com (G.V.M.); himlabafi@yandex.ru (Y.V.K.); verteb22@mail.ru (V.E.V.); vikot85@mail.ru (V.I.D.); 3 St. Petersburg Federal Research Center of the Russian Academy of Sciences, North-West Centre of Interdisciplinary Researches of Problems of Food Maintenance, Podbelskogo hwy, 7, Pushkin, 196608 St. Petersburg, Russia; tipolis@yandex.ru; 4 St. Petersburg Pasteur Institute, Federal Service for the Oversight of Consumer Protection and Welfare, 14, Mira Str., 197101 St. Petersburg, Russia; shenna1@yandex.ru; 5 Yekaterinburg Research Institute of Viral Infections, The Federal Budgetary Institution of Science “State Scientific Center of Virology and Biotechnology Vector”, The Federal Service for Supervision of Consumer Rights Protection and Human Well-Being, 23, Letnyay Str., 620030 Yekaterinburg, Russia; alexvsemenov@gmail.com; 6 CSIR-National Botanical Research Institute, Rana Pratap Marg, Lucknow 22600, India; chakrabartyd@nbri.res.in; 7 Federal Research Center N. I. Vavilov, All-Russian Institute of Plant Genetic Resources, Bolshaya Morskaya Str., 42-44, 190000 St. Petersburg, Russia; ezuev@vir.nw.ru

**Keywords:** epiphytic PGPB, *Bacillus megaterium* AFI1, *Paenibacillus nicotianae* AFI2, wheat (*Triticum aestivum* L.), Ni stress, photosynthetic pigments, antioxidant enzymes, lipid peroxidation (LPO), proline

## Abstract

The present study demonstrates the Ni toxicity-ameliorating and growth-promoting abilities of two different bacterial isolates when applied to wheat (*Triticum aestivum* L.) as the host plant. Two bacterial strains tolerant to Ni stress were isolated from wheat seeds and selected based on their ability to improve the germination of wheat plants; they were identified as *Bacillus megaterium* AFI1 and *Paenibacillus nicotianae* AFI2. The protective effects of these epiphytic bacteria against Ni stress were studied in model experiments with two wheat cultivars: Ni stress-tolerant Leningradskaya 6 and susceptible Chinese spring. When these isolates were used as the inoculants applied to Ni-treated wheat plants, the growth parameters and the levels of photosynthetic pigments of the two wheat cultivars both under normal and Ni-stress conditions were increased, though *B. megaterium* AFI1 had a more pronounced ameliorative effect on the Ni contents in plant tissues due to its synthesis of siderophores. Over the 10 days of Ni exposure, the plant growth promotion bacteria (PGPB) significantly reduced the lipid peroxidation (LPO), ascorbate peroxidase (APX), superoxide dismutase (SOD) activities and proline content in the leaves of both wheat cultivars. The PGPB also increased peroxidase (POX) activity and the levels of chlorophyll *a*, chlorophyll *b*, and carotenoids in the wheat leaves. It was concluded that *B. megaterium* AFI1 is an ideal candidate for bioremediation and wheat growth promotion against Ni-induced oxidative stress, as it increases photosynthetic pigment contents, induces the antioxidant defense system, and lowers Ni metal uptake.

## 1. Introduction

The contamination of soil with Ni and other heavy metals (HM) due to wastes from heavy industry and nonferrous metallurgy is a major environmental concern [1,2]. The concentration of Ni ions in polluted soils can reach 20–26 times higher levels compared to native soils [3,4]. Excess Ni accumulates in agricultural products due to the increasing pollution of agricultural lands [5,6]. Ni in high concentrations is toxic to plants, which manifests as the inhibition of plant growth, metabolic disorders, and oxidative stress [6]. High concentrations of Ni trigger the formation of excessive amounts of reactive oxygen species in plants [7], which leads to changes in antioxidant activities [8,9], disruptions of the nucleus structure [10] and antimitotic and genotoxic actions [11]. Ni in toxic concentrations disrupts water balance [12], membrane functions and carbohydrate metabolism [13], and reduces photosynthesis [14] and plant yields [15]. Ni toxicity in plants can cause Ni-induced stress through the accumulation of hydrogen peroxide and the related lipid peroxidation [16,17]. The generation of reactive oxygen species (ROS), which results in oxidative stress [18,19], is one important aspect of toxicity that ultimately induces the plant defense system [18,20,21]. Ni can both induce and inhibit the activity of antioxidative enzymes [22]. Increasing GPX and SOD activities in *Amaranthus paniculatus* [23], CAT, POD and SOD activities in *Lactuca sativa* L. [24], SOD, POD, CAT and APX activities in *Alyssum inflatum* Nyár. [25], as well as APX activities in pea leaves were reported [26], while the suppressed activity of CAT, APX, and SOD was reported in *Alyssum bertolonii* [27], *Triticum aestivum* L. [28] and *Grewia asiatica* L. [29].

PGPB may enhance the growth and tolerance of plants under Ni stress [30,31,32]. PGPB can eliminate HM toxicity via multiple mechanisms [33,34,35] changes in the pH and redox potential of the media, and the production of siderophores, polysaccharides and various antioxidant enzymes [36].

Bacteria from the genera *Bacillus* and *Paenibacillus* live both on the surface of the aboveground organs of plants and on their roots [37]. They promote plant growth, due to the production of phytohormones, siderophores, lipopeptides, polysaccharides and enzymes [38]. These bacteria are promising candidates for Ni and other kinds of HM bioremediation [2,39,40,41,42,43].

Many studies have shown that PGPB improve plant growth and plant defense systems when under Ni stress [44,45]. However, while the mechanisms of heavy metal tolerance in plants have been studied extensively, the role of PGPB in the regulation of metal translocation in plants remains undetermined [46,47]. There is also no information about the responses of the in vivo activities of the SOD, APX and POX pathways under metal excess, or the possible effects of PGPB inoculation under Ni stress.

We hypothesized that PGPB can affect the plant defense system and help plants to overcome Ni stress. The main objectives were to evaluate the effects of two epiphytic PGPB, *Bacillus megaterium* AFI1 and *Paenibacillus nicotianae* AFI2, on the growth and antioxidant response of two soft spring wheat cultivars (contrasting to Ni-tolerance) under Ni exposure.

## 2. Results

### 2.1. Bacterial Identification

The cells of bacterium AFI1 are Gram-positive, motile, and produce endospores. Their colonies are round, white, and smooth when grown on LB agar. The bacterium is catalase-positive and oxidase-negative; indole and H_2_S are not produced. The Voges—Proskauer reaction is negative. The bacterium can utilize glucose, arabinose, xylose, maltose, sucrose, sorbitol, mannitol, glycerol, lactate, citrate and butyrate. It does not contain ornithine decarboxylase, lysine decarboxylase or arginine dihydrolase. The bacterium can utilize histidine as a nitrogen source but cannot utilize alanine or proline. The bacterium AFI1 hydrolyzes casein, gelatin and aesculin, but cannot hydrolyze starch.

The cells of bacterium AFI2 are Gram-positive, motile and produce endospores. Grown on LB agar, it forms round, pink and smooth colonies. AFI2 is catalase-positive and oxidase-negative; indole and H_2_S are not produced. The Voges—Proskauer reaction is negative. The bacterium does not contain ornithine decarboxylase, lysine decarboxylase or arginine dihydrolase. It can utilize glucose, arabinose, xylose, maltose, sucrose, ribose, galactose, mannitol and alanine, but cannot utilize lactate, citrate, butyrate, histidine and glycerol. The bacterium hydrolyzes casein, gelatin and aesculin, but does not hydrolyze starch. The biochemical and physiological characteristics of the studied bacterial strains are summarized in Table S1 [48,49,50].

The strain AFI1 produced siderophores, polysaccharides and phytohormones. The strain AFI2 produced polysaccharides and more phytohormones compared to AFI1, but did not produce siderophores (Table 1).

### 2.2. 16 S rRNA Sequencing of PGPB

The sequences were submitted to the NCBI databases with accession numbers MZ468613 (1554 bp) for *Priestia megaterium* (the new species name for *Bacillus megaterium* after [51]) (strain AFI1) and MZ468614 (1552 bp) for *Paenibacillus nicotianae* (strain AFI2). Even though the bacteria *Bacillus megaterium* were recently renamed *Priestia megaterium* (Available at: https://www.ncbi.nlm.nih.gov/Taxonomy/Browser/wwwtax.cgi?id=1404 (accessed date: 26 October 2021)), in our article, we will use the previous classification, and describe the strain AFI1 as *Bacillus megaterium*.

Based on the EzTaxon-e server results of 16S rRNA gene sequence similarity, AFI1 showed the closest similarity (99%) to the *Bacillus* species, such as *Bacillus aryaabhattai* B8W22 (99.93%), *Bacillus megaterium* ATCC 14581 (99.8%), and *Bacillus zanthoxyli* strain 1433 (99.86%). Lower sequence similarities were found with *Bacillus flexus* NBRC 15715 (98.82%) and *Bacillus simplex* DSM 1321 (98.54%).

**Figure 1:**
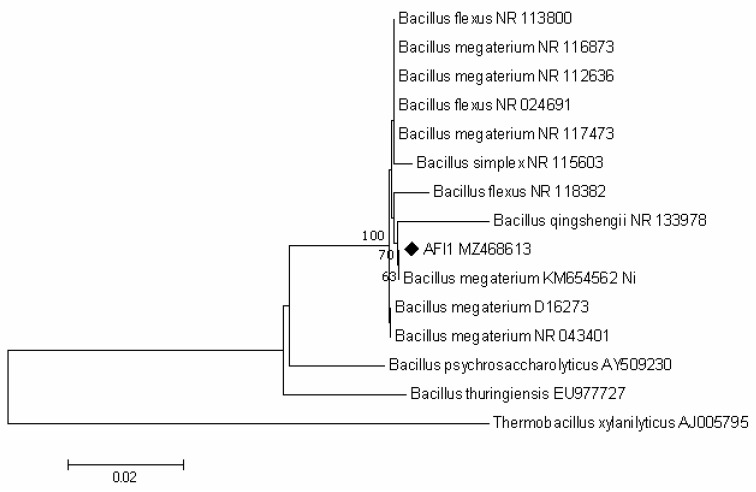
Neighbor-joining tree based on 16S rRNA gene sequence analysis showing the phylogenetic relationships of strain AFI1 and members of the genus *Bacillus.* Bootstrap values higher than 60% based on 1000 replications are shown at branching points. *Thermobacillus xylanilyticus* was used as an outgroup. Scale bar, 0.02 substitutions per nucleotide position.

The phylogenetic tree (Figure 1) shows that AFI1 (MZ468613) clustered within the genus *Bacillus* and joined with the nearest neighbor *Bacillus megaterium* KM654562, which is described as a Ni-tolerant bacterium [52].

**Figure 2:**
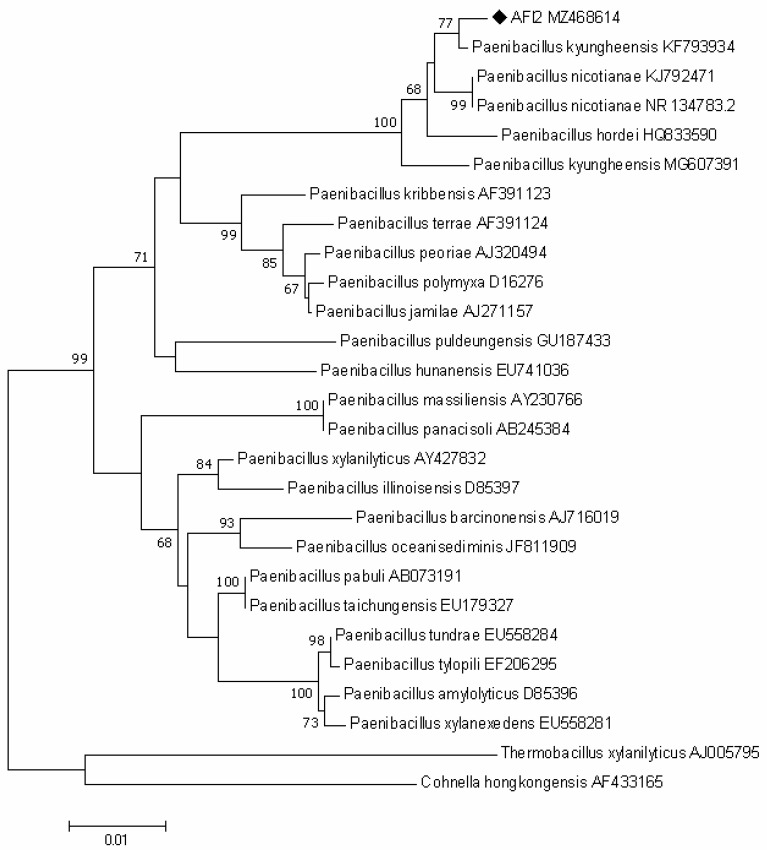
Neighbor-joining tree based on 16S rRNA gene sequence analysis showing the phylogenetic relationships of strain AFl2 and members of the genus *Paenibacillus*. Bootstrap values higher than 60% based on 1000 replications are shown at branching points. *Thermobacillus xylanilyticus* and *Cohnella hongkongensis* were used as an outgroup. Scale bar, 0.01 substitutions per nucleotide position.

After a comparison of the nucleotide sequences of 16S rRNA genes, the greatest similarity was found with *Paenibacillus nicotianae* YIM-19 (98.84%), *Paenibacillus kyungheensis* DCY-88 (98.4%) and *Paenibacillus hordei* RH-N24 (98.03%). The phylogenetic tree (Figure 2) shows that AFI2 (MZ468614) clustered with *Paenibacillus kyungheensis* KF 793934 and with *Paenibacillus nicotianae* (68%).

Based on the morphological and biochemical characteristics as well as the data of 16S rRNA genes, we identified AFI1 as *Bacillus megaterium* AFI1 (*B. megaterium* AFI1) and AFI2 as *Paenibacillus nicotianae* AFI2 (*P. nicotianae* AFI2).

### 2.3. Effect of Ni and PGPB on Wheat Growth

*B. megaterium* AFI1 and *P. nicotianae* AFI2 significantly increased the lengths of shoots sensitive to Ni stress in the cultivar Chinese spring (hereinafter Ch. Spring S) by 22.7% and 21.1%, respectively. The values of these parameters for tolerant cultivar Leningradskaya 6 (hereinafter Leningrad. 6 T) were 10% and 6.1% (Table 2, Figures S1 and S2).

However, the opposite situation for seedling roots was noted: PGPB increased the growth of roots of Leningrad. 6 T considerably more noticeably (by 55.5% and 33.9%) than those of Ch. spring S (by 9.3% and 2.8%, respectively). In general, the increase in the root FW of Ch. spring S was more pronounced and was associated with more intensive root formation after inoculation with PGPB.

The wheat Ch. spring S was more responsive to bacterial inoculation in the control variants (without a toxic Ni concentration). PGPB had greater effects on the shoot length, as well as on the shoot and root FWs, of seedlings of Ch. Spring S. The greatest root growth in Leningrad. 6 T was registered after inoculation with PGPB.

The wheat Ch. spring S was more sensitive to the presence of toxic Ni ions. The shoot length in the seedlings Ch. spring S was reduced by 36.5%, and in the seedlings of Leningrad. 6 by 17%. The root FWs of Ch. spring S were more susceptible to high concentrations of Ni (reduced by 50%), while those of cv. Leningrad. 6 decreased by 44%. Chlorosis symptoms reflecting a reduction in chl contents were observed in the middle of the vegetation period.

Inoculation with *B. megaterium* AFI 1 under Ni stress increased shoot lengths in both cultivars: Ch. spring S by 6.9% and Leningrad. 6 T by 8.6%, compared to Ni-treated plants. The effects of *P. nicotianae* AFI2 under Ni stress were more visible: the shoot lengths were increased in Ch. spring S by 13.7% and in Leningrad. 6 T by 14.7% compared to Ni-treated plants. The maximal increase in root FW was obtained after inoculation with *P. nicotianae* in AFI2 Ch. spring S compared to Ni-treated plants (without inoculation).

### 2.4. Ni Accumulation

**Figure 3:**
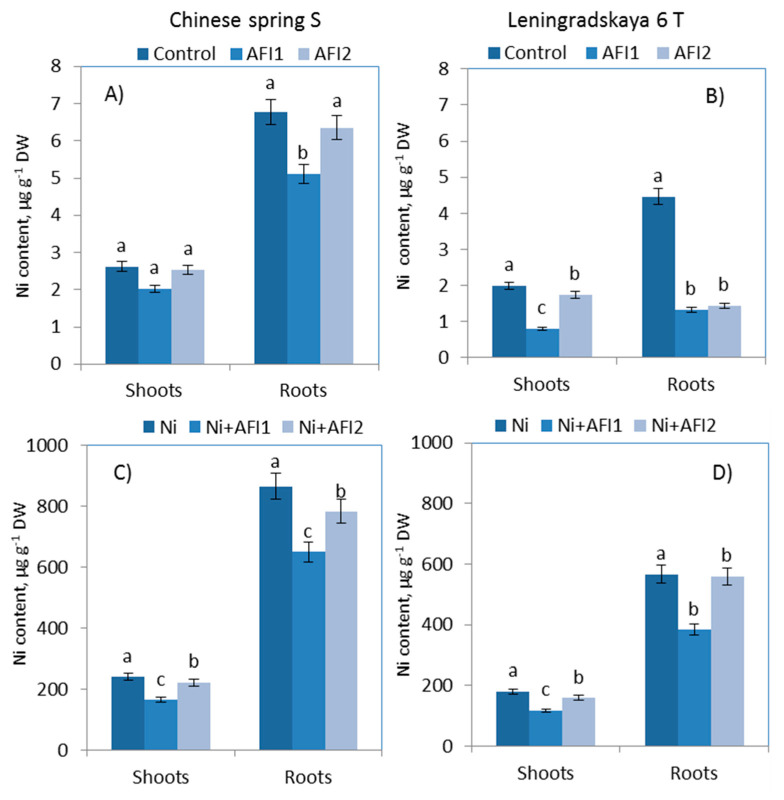
Ni content in shoots and roots of wheats: (**A**,**C**) Chinese spring S; (**B**,**D**) Leningradskaya 6 T. Different letters indicate a significant difference between the means at the probability level of *p* < 0.05, as determined by Duncan’s multiple range test.

The data shown in Figure 3 suggest that Ni ions accumulated in wheat tissues in small concentrations under control conditions (without Ni). Both inoculants decreased the Ni content in the roots and shoots of both wheat cultivars under control conditions; however, the most visible effect was registered in the roots of cv. Leningrad.6.

Under Ni exposure, the wheat cultivars accumulated varying amounts of Ni ions in their plant tissues (Figure 3). Leningrad. 6 T accumulated 34.5% less Ni in the roots and 25.2% less in the shoots compared to Ch. spring S (Figure 3).

Figure shows the results after 10 days of exposure to Ni. Control: non—inoculated wheat plants. AFI1: wheat plants inoculated with *B. megaterium* AFI1. AFI2: wheat plants inoculated with *P. nicotianae* AFI2. Ni: wheat plants grown with Ni concentration 100 µM·L^−1^. Ni + AFI1: wheat plants with Ni concentration 100 µM·L^−1^ and inoculated with *B. megaterium* AFI1. Ni + AFI2: wheat plants grown with Ni concentration 100 µM·L^−1^ and inoculated with *P. nicotianae* AFI2. Data of three independent experiments are expressed as the means of three replicates. In each experiment 30 plants of Leningrad. 6 T and 30 plants of Ch. spring S were used. DW—dry weight of shoots and roots. Bars show ±SEM and different letters show a significant difference among treatments (for shoots and roots, respectively) at the *p* < 0.05 level as determined by Duncan’s multiple range test.

The concentration of Ni ions in the wheat roots after 10 days of Ni exposure was significantly higher than in the shoots. *B. megaterium* AFI1 significantly reduced Ni content in both the roots and shoots. Leningrad. 6 T was more responsive to inoculation with *B. megaterium* AFI1: the Ni content in the shoots of Leningrad. 6 T decreased by 35.2%, and that in Ch. spring S decreased by 31.7%.

The inoculation with *P. nicotianae* AFI2 slightly reduced the Ni content in the shoots of Leningrad. 6 T, by 12%, and reduced it by 7.6% in Ch. spring S.

### 2.5. Chlorophyll and Carotenoid Content

Inoculation with *P. nicotianae* AFI2 increased the total chlorophyll (chl) level to 20.9% and that of carotenoids (car) to 30%, in Ch. spring S (Table 3). Nickel had a more pronounced negative effect on chlorophyll a (Figure 3). There were no significant changes in chl or car contents in Leningrad. 6 T when inoculated with PGPB. Leningrad. 6 T contained more chl and car compared to the sensitive Ch. spring S under control conditions. A statistically significant reduction in chl and car contents was observed under Ni stress.

**Figure 4:**
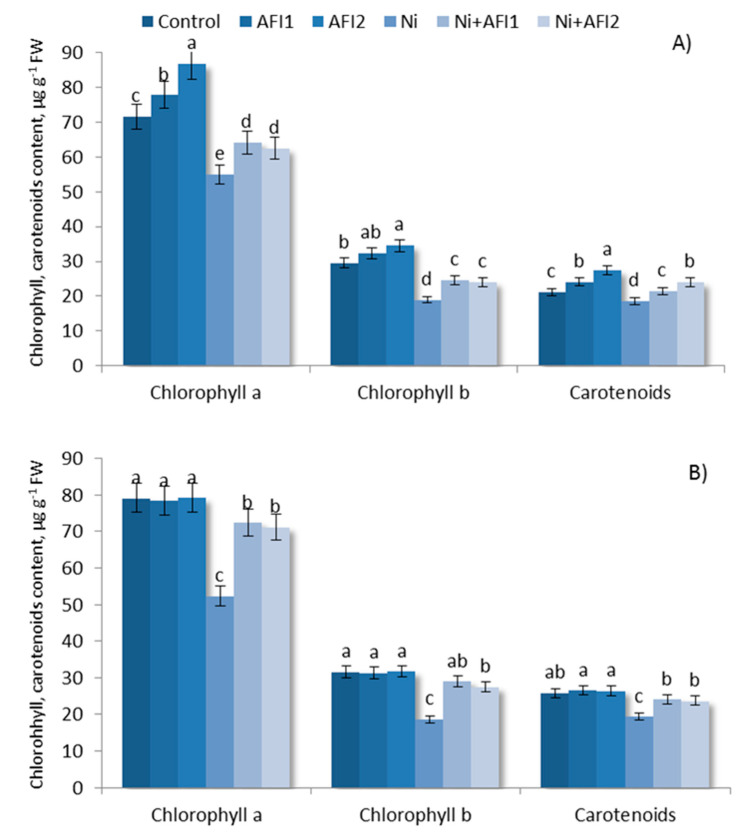
Chlorophylls and carotenoids contents in shoots of wheats: (**A**) Ch. spring S, (**B**) Leningrad. 6 T. Different letters indicate a significant difference between the means at the probability level of *p* < 0.05, as determined by Duncan’s multiple range test.

After Ni exposure, the total chl content decreased by 21.6% in Ch. spring S and by 34.3% in Leningrad. 6 T (Table 3), while the total car content decreased by 12.1% and 24.6%, respectively (Figure 4).

Figure shows the results after 10 days of exposure to Ni. Control: non—inoculated wheat plants. AFI1: wheat plants inoculated with *B. megaterium* AFI1. AFI2: wheat plants inoculated with *P. nicotianae* AFI2. Ni: wheat plants grown with Ni concentration 100 µM·L^−1^. Ni + AFI1: wheat plants with Ni concentration 100 µM·L^−1^ and inoculated with *B. megaterium* AFI1. Ni + AFI2: wheat plants grown with Ni concentration 100 µM·L^−1^ and inoculated with *P. nicotianae* AFI2. Data of three independent experiments are expressed as the means of three replicates. In each experiment 30 plants of Leningrad. 6 T and 30 plants of Ch. spring S were used. FW—fresh weight of shoots. Bars show ±SEM and different letters show a significant difference among treatments at the *p* < 0.05 level as determined by Duncan’s multiple range test.

The change in the ratio of chl a/b was more pronounced in Ch. spring S under Ni stress, where it increased by 20.8% (in Leningrad. 6 T, it increased by 12%) (Table 3). Applying PGPB under Ni stress conditions significantly increased the total chl and car contents and decreased the Ni stress load on the wheat plants.

PGPB had a more pronounced effect on the more tolerant Leningrad. 6 T under Ni stress conditions.

### 2.6. Antioxidant Enzymes Responses

The antioxidant enzymes responses after 10 days of Ni exposure and inoculation with PGPB are presented in Table 4 and Table 5. The changes in enzyme activity depending on nickel exposure time (3, 6 days) are presented in Tables S2–S6.

As shown in our experiments, Ch. spring S was characterized by a higher level of SOD and by lower CAT activity in the roots compared to Leningrad. 6 T under control conditions. PGPB reduced the CAT activity in the roots of both wheat cultivars by 14.9–25.5% in Ch. spring S and by 10.9–21.5% in Leningrad.6 T and did not significantly affect CAT activity in the shoots on day 10 of Ni exposure (Table 4). However, it was shown that the level of CAT increased during Ni exposure. After 6 and 10 days of Ni exposure, the level of CAT increased by about 30% in the shoots. The PGPB significantly reduced the level of CAT in the roots of both wheat cultivars under Ni stress but increased the CAT in the shoots (significantly for Ch. spring S and insignificantly for Leningrad. 6 T) (Table S2). Different levels of change in SOD activity under Ni stress were registered in both shoots and roots. Under Ni exposure, SOD increased significantly in the roots of both cultivars, by 19% in Ch. spring S and by 26.3% in Leningrad. 6 T. Application of PGPB under Ni stress conditions reduced the SOD level in the roots of both wheat cultivars by 16.4 and 17.8%, respectively. The SOD in the roots did not change significantly throughout Ni exposure, while the SOD in the shoots increased by 30–50% on 6th and 10th days of experiments (Table S3).

The toxic concentration of Ni ions had the most pronounced effect on the activities of both POX and APX in wheat plants. Under Ni stress, the POX increased by 88.7% in the roots of Ch. spring S and by 54.3% in the roots Leningrad. 6 T. POX activity in shoots also increased under Ni stress in both wheat cultivars. There were no significant changes in POX activity in the wheat roots after inoculation with PGPB under Ni stress. In contrast, PGPB increased the POX activity in the shoots of both cultivars under Ni stress (Table 5). The POX activity significantly increased in the shoots of both the cultivars studied (from 3rd day to 10th) in each variant. In wheat roots, POX slightly increased from the 3rd day to the 10th (Table S4). The APX significantly increased in the wheat roots and shoots in both cultivars under Ni stress. The application of PGPB under Ni exposure caused a significant decrease in APX: by 14.4–27.3% in the roots of Ch. spring S and by 9.2–13.7% in the roots of Leningrad.6 T, as compared to the variant with Ni alone. In the shoots of Ch. spring S, inoculation with bacteria under Ni stress conditions reduced the level of APX by 52.7–56.7%, while in the shoots of Leningrad. 6 T this level was reduced by 36.3–37.9% (Table 5). The APX in the roots and shoots of both wheat cultivars increased slightly during Ni exposure. Application of *B. megaterium* AFI1 and *P. nicotianae* AFI2 significantly reduced the APX in the roots and shoots of both wheat cultivars, with a greater impact on the wheat shoots (Table S5).

### 2.7. LPO

One of the metabolites of lipid peroxidation is malondialdehyde (MDA), an increase in which shows that plants are subjected to oxidative stress. Ch. spring S showed higher LPO activity compared to Leningrad. 6 T under normal conditions. Ni stress significantly increased the MDA content in both studied cultivars; while they differed in terms of Ni resistance, Ch. spring S was more affected by Ni stress.

**Figure 5:**
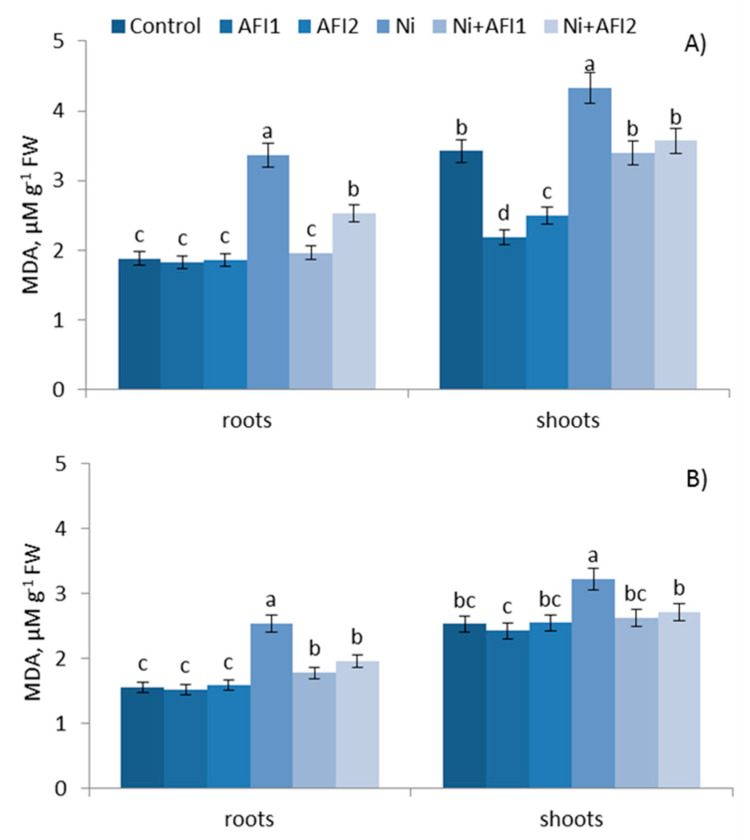
Effect of PGPB on LPO activity in wheats: (**A**) Chinese spring S, (**B**) Leningradskaya 6 T. Different letters indicate a significant difference between the means at the probability level of *p* < 0.05, as determined by Duncan’s multiple range test.

Bacteria had a similar protective effect on the MDA content in the two studied cultivars under Ni stress. PGPB under Ni stress increased membrane resistance to Ni, and reduced the MDA content in roots by 22.8–41.7% and in shoots by 15.8–21.5%. The protective effect of PGPB was more pronounced in Ch. spring S (Figure 5). The MDA was content significantly decreased in both wheat cultivars, with an increase in the duration of Ni stress (from 3 days up to 10 days). The action of PGPB was similar at all studied time points (Table S6).

Figure shows the results after 10 days of exposure to Ni. Control: non—inoculated wheat plants. AFI1: wheat plants inoculated with *B. megaterium* AFI1. AFI2: wheat plants inoculated with *P. nicotianae* AFI2. Ni: wheat plants grown with Ni concentration 100 µM·L^−1^. Ni + AFI1: wheat plants with Ni concentration 100 µM·L^−1^ and inoculated with *B. megaterium* AFI1. Ni + AFI2: wheat plants grown with Ni concentration 100 µM·L^−1^ and inoculated with *P. nicotianae* AFI2. Data of three independent experiments are expressed as the means of three replicates. In each experiment 30 plants of Leningrad. 6 T and 30 plants of Ch. spring S were used. FW—fresh weight of shoots and roots. Bars show ±SEM and different letters (a, b, c, d) show a significant difference among treatments at the *p* < 0.05 level as determined by Duncan’s multiple range test.

### 2.8. Proline

**Figure 6:**
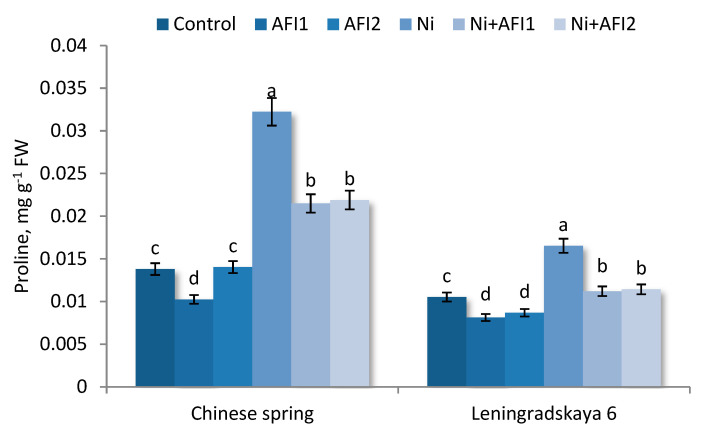
Proline content in wheat shoots of Ch. spring S and Leningrad.6 T under Ni exposure and after bacterial inoculation. Different letters indicate a significant difference between the means at the probability level of *p* < 0.05, as determined by Duncan’s multiple range test.

The content of proline was increased by 57% in the shoots of Leningrad. 6 T and by 82.8% in the shoots of Ch. spring S under Ni stress compared to control conditions. PGPB reduced proline level in the shoots of both cultivars studied under control conditions, but bacterial effect was more pronounced under the influence of Ni stress. Thus PGPB *B. megaterium* AFI1 and *P. nicotianae* AFI2 reduced proline level in the shoots of Leningrad. 6 T and Ch. spring S by 30.7–35.1% and by 18.1–25.8% as compared to the control, respectively (Figure 6). PGPB under Ni stress reduced proline content by 30.9–32.2% in shoots Leningrad. 6 T and by 25.1–26.7% in shoots Ch. spring S as compared to Ni alone.

Figure shows the results after 10 days of exposure to Ni. Control: non—inoculated wheat plants. AFI1: wheat plants inoculated with *B. megaterium* AFI1. AFI2: wheat plants inoculated with *P. nicotianae* AFI2. Ni: wheat plants grown with Ni concentration 100 µM·L^−1^. Ni + AFI1: wheat plants with Ni concentration 100 µM·L^−1^ and inoculated with *B. megaterium* AFI1. Ni + AFI2: wheat plants grown with Ni concentration 100 µM·L^−1^ and inoculated with *P. nicotianae* AFI2. Data of three independent experiments are expressed as the means of three replicates. In each experiment 30 plants of Leningrad. 6 T and 30 plants of Ch. spring S were used. FW—fresh weight of shoots. Bars show ±SEM and different letters show a significant difference among treatments at the *p* < 0.05 level as determined by Duncan’s multiple range test.

## 3. Discussion

Ni at a minimal toxic concentration (100 µML^−1^) inhibited wheat growth and reduced plant biomass (Table 2). Plant growth inhibition under Ni stress is associated with the disturbance of transpiration and water balance [53,54], with a decreased cell division rate [55] and the disruption of nutrient absorption [36,39,56]. PGPB significantly increased plant growth both under control conditions and under Ni stress, with a more visible effect of *P. nicotianae* AFI2.

Ni stress increased the MDA content in both wheat cultivars (Figure 5), and this is associated with the cellular damage caused by the excess ROS production and the lower capacity for ROS elimination in the defense system, leading to the formation of MDA. The tolerant Leningrad. 6 T, in contrast with the sensitive cultivar, was characterized by a high level of POX and low LPO. Leningrad. 6 T showed greater growth and a lower Ni accumulation compared to Ch. spring S (Figure 3). Similarly low levels of LPO have been shown in wheat genotypes tolerant to drought [57], salinity [58] and water [59] stresses. The lower LPO in Leningrad. 6 T was accompanied by higher CAT and POX levels and lower SOD and APX activities (Table 4 and Table 5). However, the activities of SOD and APX were higher in the Ni-tolerant maize hybrid Pioneer [60]. While different genotypes of the same plant species have different levels of activity of antioxidant enzymes, tolerant genotypes are characterized by a high level of enzyme activity and a low level of LPO [18,44,57]. PGPB reduced the LPO in the roots and shoots of both wheat cultivars, protecting the plant membranes from destructions. It was also found that *Bacillus subtilis* decreased the LPO in wheat plants under abiotic stress [61,62].

In our study, different SOD responses to nickel stress in wheat roots and shoots have been observed. Under Ni stress, the SOD increased in the roots and decreased in the shoots of wheat plants in our experiments (Table 4). Differences have been identified in the level of TaSOD expression between wheat roots and leaves in response to salt stress [63]. However, there is evidence that Ni decreases SOD activities in both shoots and roots compared to the control [54]. It is possible that the differences in SOD reactions in the roots and leaves described in our experiments and in earlier studies [54] are determined by the characteristics of wheat cultivars. The roots of sessile plants are the first organs to encounter heavy metals, and thus, roots have been showing more severe effects in response to a stressor, leading to high SOD activities.

HM increased the activity of SOD in plants. The exposure of wheat plants to increased concentrations of municipal solid waste compost containing heavy metals such as Ni, Pb, Cu, and Zn stimulated the activity of SOD, providing tolerance to the plant under adverse conditions [64]. Metal stress has been reported to enhance the expressions of SOD genes (Fe-SOD, Cu/Zn-SOD) in soybean seedlings [65].

Ni increased the levels of proline and antioxidant enzymes such as SOD in *Cicer arietinum*, while inoculation with the PGPB *Pseudomonas aeruginosa* significantly reduced the level of these stress markers [42]. Increasing concentrations of Ni caused exponential increases in proline content in water lettuce plants [66].

The greatest changes were recorded in POX, the activity of which considerably increased in the shoots of both cultivars. Our results are consistent with previously obtained data showing the increase in POX activity in wheat shoots [67]. The level of CAT in the roots of Ch. spring S was significantly lower than that in the roots of Leningrad. 6 T. PGPB increased the POX levels even more significantly in both cultivars (Table 5).

PGPB significantly reduced the APX in roots and shoots and the CAT in the roots of both cultivars under Ni stress. In wheat, a mutant line with reduced activity of the thylakoid APX manifests a disruption of photosynthesis [68]. The activity of APX increases with an increase in the concentration of Fe and Cu in wheat seedlings [69]. Increased expressions of POD and APX genes were also recorded in perennial ryegrass in response to metal stress [69], indicating its role in H_2_O_2_ scavenging in the plant.

Antioxidant enzymes can increase or decrease in plants, depending on various abiotic stresses and the duration of stress exposure [31].

We found no change in CAT activity in wheat leaves under Ni stress. This is consistent with the results obtained for vetch plants [9]. The CAT genes respond differently to various stress conditions [70].

The balance between SODs, CATS and APXs is important for determining the intracellular level of ROS; in addition, changes in the balance of these enzymes appear to induce compensatory mechanisms [70,71].

Wheat cultivars accumulated different Ni contents in plant tissues. The tolerant Leningrad. 6 T cultivar accumulated less Ni than the sensitive cultivar (Figure 3). This finding agrees with the results obtained for tomato genotypes differing in Ni tolerance [44]. Similarly high Ni concentrations in wheat roots (828.20–1200 µg g^−1^ after exposure to Ni at concentrations of 50–100 µM) have been recorded by other authors [72,73].

Inoculation with PGPB indicated its plant growth potential as it increased plant growth and biomass in both wheat cultivars under control conditions and Ni stress. Both inoculants reduced the Ni contents in wheat plants. However, *B. megaterium* AFI1 decreased Ni content much more significantly (up to 34.5%) compared to *P. nicotianae* AFI2. This significant reduction in nickel is due to siderophores, which are known to bind nickel, making it inaccessible to plants [32,74,75,76,77].

Along with chelates, bacteria improve plant growth through the production of phytohormones. There are data indicating that the application of auxins, gibberellins and cytokinins generally leads to a reduction in Ni concentration in the shoots of *Alissum* and *Noccaea* species [78].

Both PGPB studied produced auxins, abscisic acid and gibberellins; however, *P. nicotianae* AFI2 was found to be a more effective producer. The effect of *P. nicotianae* AFI2 on wheat shoot growth was more visible (Table 2), while it reduced the Ni content in the plant to a much lower degree compared to *B. megaterium* AFI1 (up to 12%). This positive effect of *P. nicotianae* AFI2 was induced by the more active production of phytohormones and the nitrogen-fixing activity. *P. nicotianae* AFI2 possessed 2.8-fold greater ABA activity compared to *B. megaterium* AFI1 (Table 1). ABA signaling affects plants’ gibberellin and auxin signaling pathways and controls lateral root development under stress conditions [79]. ABA also regulates water misbalance in plants by controlling stomatal closure and stress signal transduction pathways [80]. Therefore, ABA-producing *P. nicotianae* AFI2 could improve the water misbalance caused by Ni stress. It was also found that ABA increased chlorophyll content; therefore, ABA-synthesizing bacteria may be responsible for the increase in chlorophyll content observed in our experiments [81].

One of the effects of nickel exposure is a reduction in the amount of iron in plants, which directly affects the photosynthetic pigments [82].

The photosynthetic pigments were increased in inoculated sorghum plants under HM stress by beneficial strains *Alcaligenes faecalis* MG257493.1, *Bacillus cereus* MG257494.1 and *Alcaligenes faecalis* MG966440.1 [83].

The toxic concentrations of Ni ions damage mesophyll cells, epidermal tissues, thylakoid membranes and chloroplast structure, contributing to the inhibition of photosynthetic pigment function in plants [13,84]. Chl and carotenoids are indirect biochemical indicators of photosynthetic activity [85]. The chl a/b ratio is expected to increase when the leaf N content decreases [86,87].

As shown in our experiments, Ni contributed to the change in the chlorophyll a/b ratio, significantly reducing the level of total chl, and mainly chlorophyll b (Table 3). The chl a/b ratio increased more noticeably in leaves of Ch. spring S, indicating the adverse effects of Ni on nitrogen content in plant leaves. These data confirm the findings of other researchers [54]. The chlorophyll a/b ratio increased by 14% in wheat leaves after exposure to 50 µM Ni for 7 days, with the content of chlorophyll b decreasing more significantly than that of chlorophyll a [54]. It is assumed that toxic concentrations of Ni ions damage mesophyll cells, epidermal tissues, thylakoid membrane and chloroplast structure, which contributes to the inhibition of photosynthetic pigments’ function in plants [8].

PGPB inoculation significantly increased the contents of chlorophylls a and b and carotenoids under Ni stress, and reduced the ratio of chlorophylls a/b, perhaps due to the enhanced uptake of mineral nutrients that stimulate the photosynthetic and antioxidant enzymes’ activities [88].

Chl content was also increased after the bacterial inoculation of tomato and maize under Ni stress [44,60,89]. Carotenoids and phenols may cause accumulations of H_2_O_2_ and active molecular oxygen, thus protecting against oxidative damage [90].

Further research in this direction is necessary. The complete elucidation of the mechanisms within the influence of *B. megaterium* AFI1 and *P. nicotianae* AFI2 on plants under Ni stress, including an analysis of the immobilizing metabolites released by these PGPB, is a promising direction of further studies.

## 4. Materials and Methods

### 4.1. Screening and Isolation of Ni-Resistant PGPB

PGPB bacteria were isolated from seeds of spring wheat cv. Leningrad. 6 T. Flasks containing 100 mL of sterile phosphate buffer and 10 g of wheat seeds were placed in an ultrasonic bath (Bandelin; 50 Hz) for 10 min. Then the phosphate buffer solution containing microorganisms washed from the grains was serially diluted. Then, 0.1 mL of various dilutions was inoculated on Petri dishes containing LB (Luria Bertani, Sigma-Aldrich, St. Louis, USA) agar medium with an addition of Ni cations at the concentration 100 µM·L^−1^ Ni^2+^. Then, the bacterial isolates were screened for PGP activity via the method described earlier [91] with some modifications. Sterile wheat seeds were dipped for 10 min into bacterial cultures containing 10^6^ cells per mL. The seeds were placed in sterile Petri dishes (with filter paper and 10 mL of sterile water) with three replicates at 28 °C for 4 days. The lengths of seedlings (mm) of the treated and untreated (control) variants were measured. The two strains, AFI1 and AFI2 that showed the greatest plant growth promotion activity were selected for further study.

### 4.2. Bacterial Physiological and Biochemical Characteristics

The Gram reaction was determined using the Gram-staining method with the help of a bioMe’rieux Gram-staining kit. Cell morphology was examined by light-microscopy under a Zeiss light microscope (Zeiss, Berlin, Germany with 1000× magnification), while motility was assessed by the injection of bacterium into semi-liquid agar. Growth at +5 and +40 °C was assessed. NaCl (6%) tolerance was examined via growth on LB medium. Catalase activity was examined via the production of oxygen bubbles using H_2_O_2_ (3%, *v*/*v*), and the oxidase activity was detected using a commercial oxidase strip (Sigma-Aldrich, St. Louis, MO, USA). Production of acid was performed by the method in [92]. Utilization of D-glucose D-sucrose, maltose, arabinose, D-galactose, xylose, inositol, dulcitol, sorbitol, glycerol, mannitol were determined according to [91]. H_2_S production was determined according to [92]; indole production was assessed by the Ehrlich method [93]. The assimilation of 0.2% organic acids was assessed via growth on LB medium. The hydrolysis of casein, cellulose, starch and aesculin, H_2_S production, and methyl red and nitrate reduction were assayed via the method in [92]. The production of siderophores was determined as described in [94]. The formation of polysaccharides was recorded visually via the viscous consistency of the colonies. Phytohormones production was assayed after the growth of PGPB on tryptic soy broth on the 14th day of cultivation; 0.1% tryptophan was added to the growth medium for the detection of IAA.

### 4.3. Bacterial Phytohormones Assay

The concentrations of IAA, ABA, and GA3 in the extract were determined using the high-performance liquid chromatograph VARIAN 212 LC with a mass-selective detector (Varian 500 MS system). Detection was carried out using ES+ (electrospray) for the relevant characteristic ions. To determine phytohormones, 50 mL of liquid culture (and 50 mL sterile liquid medium, used as a control) was taken and centrifuged at a speed of 3000–5000 rpm for 5 min. The supernatant was drained into a dividing funnel. The precipitate was shaken twice with 30 mL of distilled water and centrifuged after combining the supernatant in a dividing funnel. The combined supernatant in the dividing funnel was acidified with a 10% solution of acetic acid to a pH of 2, after which phytohormones were extracted three times with 10 mL of ethyl acetate. The upper ethyl acetate layer was drained through anhydrous sodium sulfate and evaporated until dry on a rotary evaporator at a temperature of no more than 40 °C. The residue in the distillation flask was washed off with 2 mL of deionized water. The extraction was performed three times. Chromatography was carried out in the gradient mode (phase A, methanol + 0.1% formic acid; phase B, deionized water +0.1% formic acid). The chromatographic system used a Cosmosil C18 4.6 ID × 150 mm column.

The chromatograph was calibrated using the SIGMA-ALDRICH internal standards for pure hormone substances. The identification of hormones was carried out in the mass–mass mode.

### 4.4. 16 S rRNA Sequencing of PGPB

The genomic DNA of strains was isolated using the DNA isolation kit (AmpliPrime Ribo-Prep, Moscow, Russia). We performed 16S rRNA sequencing for AFI1 and AFI2 bacteria. The 1.5-kilobase partial sequence of the 16S rRNA gene was amplified using a polymerase chain reaction (PCR) and universal *Eubacteria*-specific primers: 8F (5′-AGAGTTTGATCCTGGCTCAG-3′) and 1541R (5′-AAGGAGGTGATCCAGCCGCA-3′). PCR experiments were conducted according to the protocol described in [95]. The PCR product was sequenced using the two primers given above and the following set of two forward and two reverse primers: 805R (5′-GGACTACCAGGGTATCTAATCCC-3′), 515R (5′-GWATTACCGCGGCKGCTG-3′), 508F (5′-AACTACGTGCCAGCAGC-3′), 908F (5′-AAACTCAAAGGAATTGACGG-3′). The numbers given in parentheses refer to the nucleotide positions of the primers and correspond to those in the 16S rDNA of *Escherichia coli*. Applied Biosystems’ 3500 Genetic Analyzer (Applied Biosystems, Foster City, CA, USA) was used for the sequence analysis. Electropherograms were analyzed using the sequencing analysis software SeqScape (Applied Biosystems, Foster City, CA, USA), and compared with the reference sequences in GenBank using BLASTN (NCBI).

### 4.5. Experimental Design

Seeds of soft spring wheat *Triticum aestivum* L. cv. Chinese spring S (sensitive to high concentrations of Ni) were obtained from N.I. Vavilov All-Russian Institute of Plant Genetic Resources (St. Petersburg, Russia), while seeds of soft spring wheat *Triticum aestivum* L. cv. Leningradskaya 6 T (tolerant to high concentrations of Ni) were obtained from Leningrad Scientific Research Institute of Agricultural Science “Belogorka” (St. Petersburg, Leningrad Region, Russia).

Wheat plants were grown on Knop solution containing CaNO_3_ (1 g), KH_2_PO_4_ (0.25 g), MgSO_4_·7H_2_O (0.25 g), KCl (0.125 g) and FeCl_3_ (0.0125 g per 1 L). The minimal inhibiting concentration of 100 µM/L of Ni cations was used for Ni-stressed variants. The seeds were sterilized in 70% ethyl alcohol for 60 s, washed with tap water, and then placed into 2% sodium hypochlorite solution for 15 min and rinsed in sterile water 7 times. The wheat seeds were germinated for 3 days, after which the seedlings were placed in vessels with Knop solution. The PGPB were grown at 28 °C for 48 h at 140 rpm in a broth of Luria–Bertani (LB) medium in a rotatory shaker. The bacterial concentration was about 10^8^ cells, according to the 0.5 McFarland Turbidity Standard (monitored at OD 600, using spectrophotometer Model Spekol 1500, Jena, Germany). Next, bacterial cells were centrifuged at 3900× *g* for 5 min; pelleted bacteria were rinsed in 10 mM MgSO_4_ and diluted to a concentration of 10^5^ cells per 1 mL of Knop solution. The final PGPB concentrations were monitored by counting the bacterial colonies grown on LB-agar medium. Then, 10 mL of bacterial suspension was added to the vessels with Knop solution. The final concentration of PGPB was 5–8 × 10^5^ cells per 1 mL of Knop solution. The vessels with wheat seedlings were placed on an installation for vegetation experiments. The vegetation was maintained in a 16-h photoperiod (16 h of light and 8 h of darkness) under a constant temperature of 25 °C during the day and 18 °C at night, with a light intensity of 23–25 klx per m^2^. The vegetation periods lasted for 3, 6, and 10 days with Ni exposure and inoculation with PGPB. After the end of each vegetation period, the plants were taken to assess their morphological and biochemical parameters. The experiments were carried out according to the following scheme: Control—non-inoculated wheat plants grown in Knop medium. AFI1—wheat plants grown in Knop solution and inoculated with *B. megaterium* AFI1, at a concentration of 5–8 × 10^−5^ cells·mL^−1^. AFI2—wheat plants grown in Knop solution and inoculated with *P. nicotianae* AFI2 at a concentration of 5–8 × 10^−5^ cells·mL^−1^. Ni—wheat plants grown in Knop solution with minimal toxic Ni concentration (100 µM·L^−1^). Ni + AFI1—wheat plants grown in Knop solution with minimal toxic Ni concentration (100 µM·L^−1^) and inoculated with *B. megaterium* AFI1 at a concentration of 5–8 × 10^−5^ cells·mL^−1^. Ni + AFI2—wheat plants grown in Knop solution with Ni concentration 100 µM·L^−1^ and inoculated with *P. nicotianae* AFI2 at a concentration of 5–8×10^−5^ cells·mL^−1^. Each variant was carried out in three biological replicates with 50 seedlings per replicate after 3 days and 30 seedlings per replicate after 6 and 10 days. The morphological parameters were measured after 10 days of Ni exposure. All experiments were repeated triplicate. We performed three biological replicates with 30 seedlings per replicate of Leningrad. 6 T and 22 seedlings per replicate of Ch. spring S.

### 4.6. Determination of Ni

The content of Ni in shoots and roots of the wheat was determined using atomic absorption spectrometry (SpektrAA 300, Varian, Mulgrave, Australia) after the wet digestion of oven-dried tissue in HNO_3_:HClO_4_ (4:1, *v*/*v*) solution at 140 °C. The flasks were placed in a boiling water bath for 3 h. The solution was cooled down and filtered, and Ni content was determined in the filtrate. Measurements were made following the instructions for the measuring device.

### 4.7. Chlorophyll and Carotenoids Analysis

For the determination of photosynthetic pigment concentrations, 0.2 g of fresh leaves were cut and placed in tubes containing 10 mL of 80% acetone for 24 h at 20 °C. The tubes were centrifuged at 20,000× *g* for 20 min, and the absorbance of the resulting supernatant was measured at 663, 645 and 470 nm using a spectrophotometer (Model Spekol 1500, Germany). The contents of chlorophyll a, chlorophyll b, and total carotenoid were calculated after [96].

### 4.8. Extraction of Proteins and Enzymes

The plant material (0.5 g of raw leaves) was ground on ice in a mortar with a small amount (1.5–2 mL) of 0.1 M phosphate buffer (pH 7.8) and with the addition of glass sand. After that, the homogenate was centrifuged at 15,000× *g* for 15 min at 4 °C. The protein levels and the enzyme activities were measured in the supernatant (a crude extract of leaves). The total soluble protein was determined as described in [97], while bovine serum albumin (BSA) was used as the standard (Figure S3).

### 4.9. Lipid Peroxidation (LPO)

The LPO level was measured as malondialdehyde MDA content, which is an LPO product [98]. The plant material (0.3 g of fresh leaves) was homogenized and filtered.

The reaction medium consists of 0.3 mL plants homogenate, 3 mL 1% H_3_PO_4_, 1 mL 0.6% TBA aqueous solution. The homogenate was put in a water bath at 95–100 °C for 60 min, after which the samples were cooled down. Then 4 mL of n-butanol were added and centrifuged for 10 min at 10.000× *g*. The absorbance of butanol extract was measured at 532 nm and 600 nm using the spectrophotometer Model Spekol 1500, Jena, Germany. The concentration of TBA-reactive products was expressed in MDA µM g^−1^ FW.

### 4.10. Superoxide Dismutase (SOD)

An assay of SOD activity (EC1.1.5.1.1) was performed as described in [99]. The reaction mixture at a volume of 3 mL contained 50 mM of sodium phosphate buffer (pH 7.8), 10 mM of methionine, 1.17 mM of riboflavin, 56 mM of nitro blue tetrazolium (NBT) and 0.1 mL of enzyme extract. One unit of SOD was defined as the amount of enzyme that inhibits NBT photo-reduction by 50% when monitored at 560 nm. The calculation was made according to the formula: SOD = lg(OA control/OA test)/(lg2∙PC). Here, OA control—optical absorbance of control sample, OA test—optical absorbance of test sample, PC—protein content. SOD activity was expressed U mg^−1^protein per minute.

### 4.11. Ascorbate Peroxidase (APX)

The APX (EC1.11.1.1) activity was assessed after [100] with the use of a 3 mL reaction solution containing 50 mM sodium phosphate buffer (pH 7.0), 0.5 mM L-ascorbate, 0.1 mM H_2_O_2,_ 0.1 mM Na-EDTA and 0.4 mL of the enzyme extract. The absorbance was measured at 290 nm. For the calculation of the activity, the extinction coefficient of 2.8 mM^−1^ cm^−1^ was employed. APX activity was expressed in nM ascorbate oxidized per minute per mg protein.

### 4.12. Catalase (CAT)

The CAT (EC1.11.1.6) was measured via the method of Aebi [101]. This method is based on the effect of catalase on hydrogen peroxide and the measurement of the ultraviolet absorption of hydrogen peroxide at 240 nm. The reaction mixture at a volume of 3 mL contained 0.1 M of sodium phosphate buffer (pH 7.0), 2 mM of H_2_O_2_ and 0.2 mL of enzyme extract. For the calculation of the activity, the extinction coefficient of 0.036 mM^−1^ cm^−1^ was employed. CAT activity was expressed in µM of H_2_O_2_ per mg^−1^ protein min^−1^.

### 4.13. Guaiacol Peroxidase (POX)

The activity of guaiacol peroxidase (EC 1.11.1.7) was measured using a modified version of the method in [102]. Fresh leaves (0.5 g) were ground with 50 mL of 0.1 M phosphate buffer (pH 7.0), and the homogenate was centrifuged for 20 min at 15,000× *g* at 4 °C. The assay mixture contained 0.1 M phosphate buffer (pH 7.0), 0.5 mL of guaiacol, 0.5 mL of H_2_O_2_ (0.33%) and 0.5 mL of the enzyme extract. The increase in absorbance, which was due to the oxidation of guaiacol to tetraguaiacol (e = 26.6 mM^−1^ cm^−1^), was measured for 3 min at 470 nm. POX activity was expressed in units, each representing the absorbance value per second per g^−1^ FW.

### 4.14. Proline

The content of proline was determined following Bates’s method [103]. Fresh leaves (1 g) were homogenized in 8 mL of 3% sulfosalicylic acid and centrifuged for 20 min at 15,000× *g*. Then 2 mL of supernatant was mixed with 2 mL of acid ningydrin reagent and 2 mL of glacial acetic acid. The mixture was heated in a water bath at 100 °C for 1 h. The reaction was arrested by the immersion of the test tubes in cold water (20 °C). The reaction mixture was extracted with toluene (4 mL). Absorption was measured at 520 nm, with toluene used as a control. The calibration curve was used to determine the concentration of proline in the supernatant (Figure S4).

### 4.15. Statistical Analysis

To assess the wheat growth parameters, independent experiments were performed three times with 30 seedlings of Leningrad. 6 (*n* = 90) and 22 seedlings of Ch. spring (*n* = 66). We also used three biological replicates, with 30 seedlings per replicate, to assess the biochemical parameters of plants (*n* = 9). The data were statistically evaluated using STATISTICA-11 and subjected to a two-way analysis of variance (ANOVA). The mean values are shown as error bars representing standard errors of the means in all the figures. The data are presented as average mean ± standard error (SEM). Duncan’s multiple range test was performed to determine significant differences between individual means.

## 5. Conclusions

These results clearly show that epiphytic PGPB *B. megaterium* AFI1 and *P. nicotianae* AFI2 could significantly increase the growth and decrease the Ni content in wheat cultivars Ch. spring S and Leningrad. 6 T exposed to 100 µM L^−1^ Ni. Both inoculants produced auxins, abscisic acid and gibberellins, and *B. megaterium* AFI1 produced siderophores. Even though *B. megaterium* AFI1 decreased the Ni content more significantly (up to 34.5%), compared to *P. nicotianae* AFI2, the latter had a more visible effect on wheat growth under Ni stress, due to the production of large amounts of phytohormones and the nitrogen-fixing activity. Both inoculants elevated chl a, chl b and car contents, and decreased the MDA and proline contents in the leaves of both cultivars under Ni stress. However, PGPB did not affect chl a, chl b or car contents, and insignificantly reduced the MDA contents in the leaves of Leningrad. 6 T under normal conditions. This contrasts with the results for Chines spr. S. *B. megaterium* AFI1 decreased the proline contents in both cultivars under normal conditions, and especially under Ni stress conditions. While *P. nicotianae* AFI2 did not reduce the proline content in Ch. spring under normal conditions, this bacterium significantly reduced the proline content under Ni stress conditions in both cultivars. PGPB decreased the CAT, APX and SOD levels in the roots and shoots of both cultivars, increased the POX in the shoots and did not affect the POX in the roots under Ni stress. Even though the activity of antioxidant enzymes was significantly increased by Ni stress, the inoculated plants showed the best growth performances, and the activities of most antioxidant enzymes were reduced. *B. megaterium* AFI1 may be used for growing environmentally friendly food products in soils with a low level of Ni and other HM contamination. Even though our results open up new directions for phytostabilization technology, this strain must be tested in greenhouse and field experiments.

## Figures and Tables

**Table 1:** Phytohormones and siderophores productions by PGPB studied.

	IAA, µg/L	ABA, µg/L	GAS3 µg/L	Siderophores
AFI1	33.6 ± 0.36	14.4 ± 0.41	2.90 ± 0.28	+
AFI2	61.7 ± 0.35	40.4 ± 0.28	40.0 ± 0.82	−

**Table 2:** Effect of toxic Ni concentration and inoculation with PGPB on wheat length and fresh weight (FW).

	Shoots		Roots		Seedlings
	Length, mm	FW, mg	Length, mm	FW, mg	FW, mg
	Chinese spring S				
Control	282.9 ± 29.50 ^b^	26.9 ± 4.18 ^c^	90.7 ± 5.61 ^ab^	12.6 ± 1.97 ^c^	39.5 ± 5.74 ^c^
AFI1	347.3 ± 15.20 ^a^	38.5 ± 2.02 ^a^	99.1 ± 5.88 ^a^	21.5 ± 2.41 ^a^	59.9 ± 4.00 ^a^
AFI2	342.7 ± 13.43 ^b^	35.0 ± 2.58 ^b^	83.2 ± 3.56 ^b^	18.7 ± 2.13 ^b^	53.7 ± 3.84 ^b^
Ni	157.9 ± 8.20 ^d^	12.3 ± 0.99 ^e^	56.4 ± 6.20 ^c^	6.3 ± 1.08 ^d^	18.6 ± 1.90 ^e^
Ni + AFI1	168.9 ± 11.45 ^cd^	12.8 ± 0.88 ^e^	62.1 ± 4.07 ^c^	7.0 ± 0.74 ^d^	19.8 ± 1.37 ^de^
Ni + AFI2	179.6 ± 8.18 ^c^	15.1 ± 1.41 ^d^	59.3 ± 5.52 ^c^	7.6 ± 0.79 ^d^	22.8 ± 2.01 ^d^
	Leningradskaya 6 T				
Control	268.8 ± 4.66 ^b^	23.6 ± 1.50 ^b^	99.5 ± 4.09 ^c^	8.4 ± 0.62 ^b^	32.0 ± 1.72 ^b^
AFI1	295.8 ± 4.96 ^a^	30.0 ± 1.16 ^a^	154.7 ± 11.70 ^a^	11.4 ± 1.61 ^a^	41.3 ± 2.39 ^a^
AFI2	285.3 ± 4.64 ^a^	31.8 ± 0.81 ^a^	133.2 ± 6.05 ^b^	11.1 ± 1.05 ^a^	43.0 ± 1.46 ^a^
Ni	223.1 ± 7.03 ^d^	12.0 ± 0.83 ^d^	49.8 ± 2.55 ^d^	4.7 ± 0.29 ^c^	16.6 ± 0.92 ^d^
Ni + AFI1	242.3 ± 6.14 ^c^	15.2 ± 0.58 ^c^	56.7 ± 3.59 ^d^	5.0 ± 0.30 ^c^	20.3 ± 0.66 ^e^
Ni + AFI2	255.9 ± 6.99 ^bc^	15.2 ± 0.64 ^c^	60.9 ± 3.09 ^d^	4.9 ± 0.42 ^c^	20.1 ± 0.76 ^c^

Control: non—inoculated wheat plants. AFI1: wheat plants inoculated with *Bacillus megaterium* AFI1. AFI2: wheat plants inoculated with *P. nicotianae* AFI2. Ni: wheat plants grown with Ni. Ni + AFI1: wheat plants grown with Ni and inoculated with *B. megaterium* AFI1. Ni + AFI2: wheat plants grown with Ni and inoculated with *P. nicotianae* AFI2. The bars are the means of three biological replicates with 30 seedlings of Leningrad. 6 T and 22 seedlings of Ch. spring per replicate. FW—fresh weight of shoots and roots. Bars show ±SEM and different letters show a significant difference among treatments (for shoot and roots, respectively) at the *p* < 0.05 level, as determined by Duncan’s multiple range test. Different letters indicate a significant difference between the means at the probability level of *p* < 0.05, as determined by Duncan’s multiple range test.

**Table 3:** Effects of PGPB *Bacillus megaterium* AFI1 and *Paenibacillus nicotianae* AFI2 on total chlorophyll content and chlorophyll ratio in wheat plants under Ni stress.

Treatment	Chlorophyll a/b Ratio	Total Chlorophyll, µg g^−1^ FW
	Chinese spring S	
Control	2.4	101.31 ± 1.94 ^c^
AFI1	2.4	110.16 ± 3.23 ^b^
AFI2	2.5	121.35 ± 2.66 ^a^
Ni	2.9	74.07 ± 2.88 ^e^
Ni + AFI1	2.6	88.78 ± 2.00 ^d^
Ni + AFI2	2.6	86.53 ± 3.15 ^d^
	Leningradskaya 6 T	
Control	2.5	110.79 ± 3.01 ^a^
AFI1	2.5	109.81 ± 3.19 ^a^
AFI2	2.5	110.90 ± 2.89 ^a^
Ni	2.8	71.05 ± 2.65 ^c^
Ni + AFI1	2.5	101.37 ± 2.79 ^b^
Ni + AFI2	2.6	98.61 ± 2.00 ^b^

Control: non—inoculated wheat plants. AFI1: wheat plants inoculated with *B. megaterium* AFI1. AFI2: wheat plants inoculated with *P. nicotianae* AFI2. Ni: wheat plants grown with Ni. Ni + AFI1: wheat plants grown with Ni and inoculated with *B. megaterium* AFI1. Ni + AFI2: wheat plants grown with Ni and inoculated with *P. nicotianae* AFI2. Data of three independent experiments are expressed as the means of three replicates. In each experiment 30 plants of Leningrad. 6 T and 30 plants of Ch. spring S were used. FW—fresh weight of shoots. Bars show ±SEM and different letters show a significant difference among treatments at the *p* < 0.05 level as determined by Duncan’s multiple range test.

**Table 4:** Effects of PGPB on CAT (µM H_2_O_2_ mg^−1^ protein min^−1^) and SOD (U mg^−1^ protein min^−1^) activities under Ni stress.

Treatment	CAT in Roots µM H_2_O_2_ mg^−1^ Protein min^−1^	CAT in Shoots µM H_2_O_2_ mg^−1^ Protein min^−1^	SOD in Roots U mg^−1^ Protein min^−1^	SOD in Shoots U mg^−1^ Protein min^−1^
Chinese spring S				
Control	299.9 ± 16.9 ^a^	566.2 ± 25.6 ^a^	0.285 ± 0.013 ^d^	0.353 ± 0.018 ^a^
AFI1	255.1 ± 15.4 ^b^	572.7 ± 29.4 ^a^	0.298 ± 0.014 ^cd^	0.256 ± 0.013 ^d^
AFI2	223.3 ± 9.4 ^c^	570.2 ± 24.2 ^a^	0.313 ± 0.013 ^bc^	0.313 ± 0.014 ^b^
Ni	307.7 ± 14.9 ^a^	540.5 ± 26.0 ^a^	0.339 ± 0.014 ^a^	0.295 ± 0.014 ^c^
Ni + AFI1	250.4 ± 12.7 ^b^	570.8 ± 34.1 ^a^	0.331 ± 0.016 ^a^	0.238 ± 0.014 ^e^
Ni + AFI2	222.8 ± 8.9 ^c^	563.4 ± 20.0 ^a^	0.324 ± 0.016 ^ab^	0.244 ± 0.015 ^de^
Leningradskaya6 T				
Control	550.5 ± 18.7 ^a^	501.1 ± 18.6 ^a^	0.137 ± 0.005 ^c^	0.140 ± 0.005 ^a^
AFI1	490.7 ± 14.7 ^b^	499.6 ± 13.1 ^a^	0.157 ± 0.007 ^ab^	0.135 ± 0.005 ^a^
AFI2	432.0 ± 13.4 ^c^	509.2 ± 18.2 ^a^	0.164 ± 0.007 ^ab^	0.146 ± 0.006 ^a^
Ni	285.9 ± 15.3 ^d^	491.5 ± 15.5 ^a^	0.173 ± 0.006 ^a^	0.115 ± 0.004 ^b^
Ni + AFI1	257.6 ± 16.9 ^e^	490.0 ± 19.9 ^a^	0.149 ± 0.005 ^bc^	0.097 ± 0.006 ^c^
Ni + AFI2	220.8 ± 15.7 ^f^	501.0 ± 25.9 ^a^	0.159 ± 0.011 ^ab^	0.102 ± 0.005 ^b^

Control: non—inoculated wheat plants. AFI1: wheat plants inoculated with *B. megaterium* AFI1. AFI2: wheat plants inoculated with *P. nicotianae* AFI2. Ni: wheat plants grown with Ni concentration 100 µM·L^−1^. Ni + AFI1: wheat plants with Ni concentration 100 µM·L^−1^ and inoculated with *B. megaterium* AFI1. Ni + AFI2: wheat plants grown with Ni concentration 100 µM·L^−1^ and inoculated with *P. nicotianae* AFI2. Data of three independent experiments are expressed as the means of three replicates. In each experiment 30 plants of Leningrad. 6 T and 30 plants of Ch. spring S were used. Bars show ±SEM and different letters show a significant difference among treatments at the *p* < 0.05 level as determined by Duncan’s multiple range test.

**Table 5:** Effects of PGPB on POX (U s^−1^ g^−1^ FW) and APX (nM ascorbate mg^−1^ protein min^−1^) activities under Ni stress.

Treatment	POX in Roots (U s^−1^ g^−1^ FW)	POX in Shoots (U s^−1^ g^−1^ FW)	APX in Roots (nM Ascorbate mg^−1^ Protein min^−1^)	APX in Shoots (nM Ascorbate mg^−1^ Protein min^−1^)
Chinese spring S				
Control	61.9 ± 2.8 ^c^	32.5 ± 1.3 ^d^	5.60 ± 0.37 ^d^	2.09 ± 0.07 ^d^
AFI1	70.6 ± 2.1 ^b^	42.1 ± 1.8 ^c^	6.51 ± 0.23 ^c^	2.41 ± 0.13 ^bc^
AFI2	75.0 ± 3.4 ^b^	42.3 ± 1.6 ^c^	6.79 ± 0.44 ^c^	2.30 ± 0.15 ^bc^
Ni	116.8 ± 4.9 ^a^	74.0 ± 3.2 ^b^	9.05 ± 0.32 ^a^	5.24 ± 0.17 ^a^
Ni + AFI1	118.8 ± 8.2 ^a^	82.3 ± 4.3 ^a^	7.75 ± 0.37 ^b^	2.48 ± 0.09 ^b^
Ni + AFI2	119.2 ± 3.7 ^a^	83.3 ± 4.3 ^a^	6.58 ± 0.28 ^c^	2.27 ± 0.07 ^c^
Leningradskaya 6 T				
Control	65.9 ± 3.7 ^c^	36.1 ± 2.3 ^d^	4.30 ± 0.24 ^d^	1.34 ± 0.06 ^e^
AFI1	72.2 ± 4.8 ^c^	38.4 ± 2.1 ^d^	4.95 ± 0.19 ^c^	1.54 ± 0.09 ^d^
AFI2	72.4 ± 3.4 ^c^	37.6 ± 2.1 ^d^	4.76 ± 0.25 ^c^	1.86 ± 0.09 ^c^
Ni	101.7 ± 5.5 ^b^	55.3 ± 2.4 ^c^	7.16 ± 0.40 ^a^	3.75 ± 0.18 ^a^
Ni + AFI1	109.3 ± 5.5 ^a^	64.3 ± 2.2 ^b^	6.50 ± 0.30 ^b^	2.39 ± 0.13 ^b^
Ni + AFI2	105.7 ± 4.1 ^ab^	68.9 ± 3.2 ^a^	6.18 ± 0.29 ^b^	2.33 ± 0.16 ^b^

Control: non—inoculated wheat plants. AFI1: wheat plants inoculated with *B. megaterium* AFI1. AFI2: wheat plants inoculated with *P. nicotianae* AFI2. Ni: wheat plants grown with Ni concentration 100 µM·L^−1^. Ni + AFI1: wheat plants with Ni concentration 100 µM·L^−1^ and inoculated with *B. megaterium* AFI1. Ni + AFI2: wheat plants grown with Ni concentration 100 µM·L^−1^ and inoculated with *P. nicotianae* AFI2. Data of three independent experiments are expressed as the means of three replicates. In each experiment 30 plants of Leningrad. 6 T and 30 plants of Ch. spring S were used. FW—fresh weight of shoots and roots. Bars show ±SEM and different letters show a significant difference among treatments at the *p* < 0.05 level as determined by Duncan’s multiple range test.

## Data Availability

The data presented in this study are available on request from the corresponding authors.

## Supplementary Materials

The following are available online at https://www.mdpi.com/article/10.3390/plants10112334/s1, Figure S1: Effect of *Bacillus megaterium* AFI1 and *Paenibacillus nicotianae* on growth of Ni sensitive wheat cv. Chinese Spring S after 10 days of Ni exposure, Figure S2: Effect of *Bacillus megaterium* AFI1 and *Paenibacillus nicotianae* AFI2 on growth of tolerant to Ni wheat cv. Leningradskaya 6 T after 10 days of Ni exposure. Figure S3: Protein calibration curve. Figure S4: Proline calibration curve. Table S1: Morphological, physiological and biochemical characteristics of the studied strains AFI1 and AFI2, Table S2: Effect of PGPB on CAT activity (µM H_2_O_2_ mg^−1^ protein min^−1^**)** of wheat plants under Ni stress. Table S3: Effect of PGPB on SOD (U mg^−1^protein min^−1^) activity of wheat plants under Ni stress. Table S4: Effect of PGPB on POX (U s^−1^ g^−1^ FW) activity of wheat plants under Ni stress. Table S5: Effect of PGPB on APX activity (nM ascorbate mg^−1^ protein min^−1^) of wheat plants under Ni stress. Table S6: Effect of PGPB on lipid oxidation status LPO (measured in MDA, µM g^−1^FW) of wheat plants under Ni stress.
