# Supplementary material for: Epiphytic PGPB Bacillus megaterium AFI1 and Paenibacillus nicotianae AFI2 Improve Wheat Growth and Antioxidant Status under Ni Stress
Source: Plants (Basel). 2021 Oct 29;10(11):2334. doi: 10.3390/plants10112334 (PMC8620400; doi:10.3390/plants10112334)
Supplement: Supplementary file 1 [file plants-10-02334-s001.zip › plants-1383195-supplementary.pdf]

Table S1. Morphological, physiological and biochemical characteristics of the studied strains AFI1 and AFI2.

**Characteristics**	*Bacillus flexus DSM1320* *	*Bacillus megaterium DSM32* *	**AFI1**	*Paenibacillus kyungheensis* **DCY88****	*Paenibacillus nicotianae* **YIM h-19*****	**AFI2**
Gram staining	+	+	+	+	+	+
Cell shape	Rod	Rod	Rod	Rod	Rod	Rod
Spore-forming	+	+	+	+	+	+
Morphology of colonies	Round, white, smooth	Round, white, smooth	Round, white, smooth	Round, pink, smooth	Round, yellow, smooth	Round, pink, smooth
Motile	+	+	+	+	+	+
Growth at 5^{0}C	+	+	+	+	+	+
Growth at 40^{0}C	+	+	+	-	+	+
Growth on 6% NaCl	+	+	+	-	-	-
Oxidase	+	-	-	-	-	-
Catalase	-	+	+	+	+	+
Indole production	-	-	-	-	-	-
H_2S production	-	-	-	-	-	-
Voges-Proskauer	-	-	-	-	-	-
**Utilization of**						
Glucose	+	+	+	+	+	+
Arabinose	+	+	+	-	+	+
Xylose	+	+	+	-	+	+
Maltose	+	+	+	+	+	+
Sucrose	+	-	+	+	+	+
Ribose	+	-	-	+	+	+
Galactose	+	-	-	+	+	+
Sorbitol	+	+	+	-	-	-
Mannitol	-	+	+	+	+	+
Glycerol	+	+	+	-	+	-
Lactate	+	+	+	–	n/d	-
Citrate	+	+	+	n/d	-	-
Butyrate	+	+	+	n/d	-	-
Nitrate reduction	-	+	+	-	-	-
Ornithine decarboxylase	-	-	-	-	-	-

Lysine decarboxylase	-	-	-	-	-	-
Arginine dihydrolase	-	-	-	-	-	-
Alanine	+	-	-	-	+	+
Proline	+	-	-	-	-	-
Histidine	+	+	+	-	-	-
**Hydrolysis of**						
Casein	-	+	+	+	+	+
Starch	+	-	-	+	-	-
Gelatin	n/d	n/d	+	+	+	+
Aesculin	-	+	+	+	+	+

Data of three independent experiments are expressed as the means of three replicates; Data for *Bacillus megaterium* DSM32*, for *Paenibacillus kyungheensis* DCY88** and for *Paenibacillus nicotianae* YIM h-19*** are shown in: [31], [32], [33] respectively.

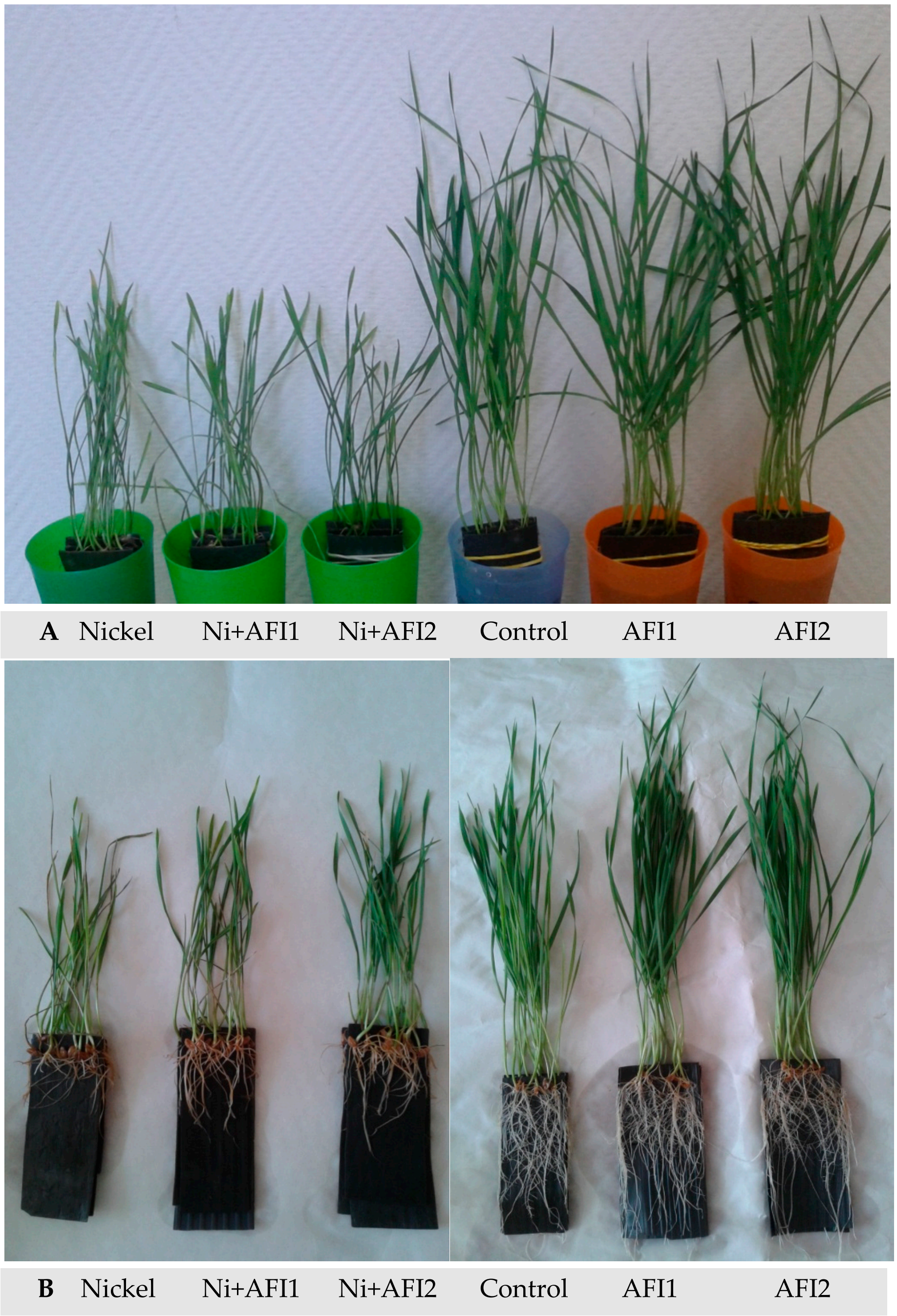

Figure S1. Effect of *Bacillus megaterium* AFI1 and *Paenibacillus nicotianae* on growth of Ni sensitive wheat cv. Chinese Spring S after 10 days of Ni exposure.

Figure 1A is representing of PGPB effect on growth of wheat shoots. Figure 1B is representing of PGPB effect on growth of wheat roots. Control: non-inoculated wheat plants. AFI1: wheat plants inoculated with *B. megaterium* AFI1 (in concentration $5\text{-}8\times10^{-5}$ cells mL^{-1}). AFI2: wheat plants inoculated with *P. nicotianae* AFI2 (in concentration $5\text{-}8\times10^{-5}$ cells mL^{-1}). Ni: wheat plants grown with Ni concentration 100 $\mu M \cdot L^{-1}$. Ni + AFI1: wheat plants with Ni concentration 100 $\mu M \cdot L^{-1}$ and inoculated with *B. megaterium* AFI1. Ni + AFI2: wheat plants grown with Ni concentration 100 $\mu M \cdot L^{-1}$ and inoculated with *P. nicotianae* AFI2. Each variant was carried out in three biological replicates with 20 seedlings per replicate.

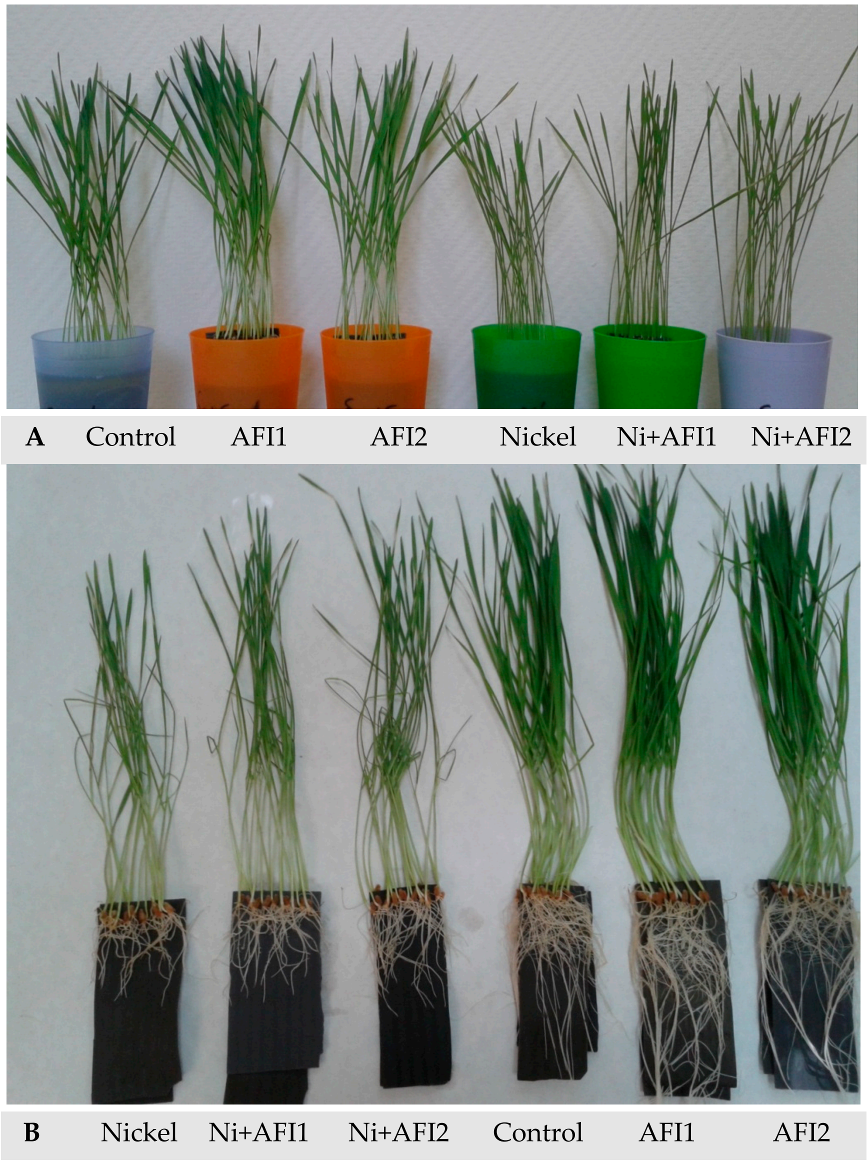

Figure S2. Effect of *Bacillus megaterium* AFI1 and *Paenibacillus nicotianae* AFI2 on growth of tolerant to Ni wheat cv. Leningradskaya 6 T after 10 days of Ni exposure.

Figure 1A is representing of PGPB effect on growth of wheat shoots. Figure 1B is representing of PGPB effect on growth of wheat roots. Control: non-inoculated wheat plants. AFI1: wheat plants inoculated with B. megaterium AFI1 (in concentration $5\text{-}8\times10^{-5}$ cells mL^{-1}). AFI2: wheat plants inoculated with *P. nicotianae* AFI2 (in concentration 5-8×10-5 cells mL^{-1}). Ni: wheat plants grown with Ni concentration 100 $\mu M \cdot L^{-1}$. Ni + AFI1: wheat plants with Ni concentration 100 $\mu M \cdot L^{-1}$ and inoculated with *B. megaterium* AFI1. Ni + AFI2: wheat plants grown with Ni concentration 100 $\mu M \cdot L^{-1}$ and inoculated with P. nicotianae AFI2. Each variant was carried out in three biological replicates with 20 seedlings per replicate.

Table S2. Effect of PGPB on CAT activity (μM H_2O_2 mg^{-1} protein min^{-1}**)** of wheat plants under Ni stress.

Experimental variants	Ni exposure time		
	3 days	6 days	10 days
	CAT in roots of cv Chinese spring S		
Control	306.0±8.6 ^{a}	292.6±11.3 ^{a}	299.9±16.9 ^{a}
AFI1	240.5±8.6 ^{b}	251.6±11.0 ^{b}	255.1±15.4 ^{b}
AFI2	227.1±17.6 ^{bc}	236.6±10.8 ^{bc}	223.3±9.4 ^{c}
Ni	304.5±7.1 ^{a}	297.4±10.2 ^{a}	307.7±14.9 ^{a}
Ni+AFI1	230.5±7.0 ^{bc}	242.6±11.7 ^{bc}	250.4±12.7 ^{b}
Ni+AFI2	215.2±8.2 ^{c}	227.3±11.3 ^{c}	222.8±8.9 ^{c}
	CAT in shoots of cv Chinese spring S		
Control	384.7±13.3 ^{a}	530.7±15.4 ^{b}	566.2±25.6 ^{a}
AFI1	378.3±15.6 ^{ab}	541.5±14.7 ^{ab}	572.7±29.4 ^{a}
AFI2	365.8±11.2 ^{abc}	555.2±19.0 ^{a}	570.2±24.2 ^{a}
Ni	339.5±12.3 ^{d}	525.7±13.8 ^{b}	540.5±26.0 ^{a}
Ni+AFI1	357.3±9.8 ^{cd}	560.7±12.7 ^{a}	570.8±34.1 ^{a}
Ni+AFI2	362.9±13.2 ^{bc}	557.1±17.3 ^{a}	563.4±20.0 ^{a}
	CAT in roots of cv Leningradskaya 6 T		
Control	536.0±12.5 ^{a}	534.1±15.9 ^{a}	550.5±18.7 ^{a}
AFI1	472.8±15.0 ^{b}	490.0±12.5 ^{b}	490.7±14.7 ^{b}
AFI2	422.4±13.8 ^{c}	441.0±10.6 ^{c}	432.0±13.4 ^{c}
Ni	253.6±12.3 ^{d}	252.9±13.0 ^{d}	285.9±15.3 ^{d}
Ni+AFI1	231.4±14.7 ^{e}	247.8±15.7 ^{d}	257.6±16.9 ^{e}
Ni+AFI2	225.5±9.8 ^{e}	234.2±15.5 ^{d}	220.8±15.7 ^{f}
	CAT in shoots of cv Leningradskaya 6 T		
Control	351.1±11.1 ^{ab}	462.0±14.2 ^{a}	501.1±18.6 ^{a}
AFI1	355.0±11.7 ^{a}	476.0±13.3 ^{a}	499.6±13.1 ^{a}
AFI2	357.9±13.8 ^{a}	477.3±12.2 ^{a}	509.2±18.2 ^{a}
Ni	332.7±12.7 ^{b}	456.7±13.5 ^{a}	491.5±15.5 ^{a}
Ni+AFI1	339.4±13.1 ^{ab}	459.7±14.1 ^{a}	490.0±19.9 ^{a}
Ni+AFI2	342.1±12.4 ^{ab}	472.3±14.5 ^{a}	501.0±25.9 ^{a}

Control: non-inoculated wheat plants. AFI1: wheat plants inoculated with *B. megaterium* AFI1 (in concentration 5-8×10^{-5} cells mL-1). AFI2: wheat plants inoculated with *P. nicotianae* AFI2 (in concentration 5-8×10^{-5} cells mL^{-1}). Ni: wheat plants grown with Ni concentration 100 μM·L^{-1}. Ni + AFI1: wheat plants with Ni concentration 100 μM·L^{-1} and inoculated with B. megaterium AFI1. Ni + AFI2: wheat plants grown with Ni concentration 100 μM·L^{-1} and inoculated with P. nicotianae AFI2.

Plant samples were analyzed after 3, 6, 10 days of Ni exposure. Each variant was carried out in three biological replicates with 50 seedlings per replicate after 3 days and with 30 seedlings per replicate after 6, 10 days. Each experiment was repeated 3 times. Bars show ± SEM and different letters show a significant difference among treatments at $p<0.05$ level as determined by Duncan's multiple range test. CAT activity was expressed in μM of H_2O_2 per mg^{-1} protein min^{-1}.

Table S3. Effect of PGPB on SOD (U mg^{-1}protein min^{-1}) activity of wheat plants under Ni stress.

Experimental variants	Ni exposure time 3 days	6 days	10 days
	SOD in roots of cv Chinese spring S		
Control	0.255±0.010 ^{d}	0.285±0.011 ^{d}	0.285±0.013 ^{d}
AFI1	0.275±0.007 ^{c}	0.291±0.011 ^{d}	0.298±0.014 ^{cd}
AFI2	0.278±0.009 ^{c}	0.316±0.007 ^{c}	0.313±0.013 ^{bc}
Ni	0.303±0.007 ^{b}	0.338±0.010 ^{a}	0.339±0.014 ^{a}
Ni+AFI1	0.315±0.009 ^{a}	0.332±0.014 ^{ab}	0.331±0.016 ^{a}
Ni+AFI2	0.310±0.010 ^{ab}	0.323±0.008 ^{bc}	0.324±0.016 ^{ab}
	SOD in shoots of cv Chinese spring S		
Control	0.174±0.007 ^{a}	0.247±0.009 ^{a}	0.353±0.018 ^{a}
AFI1	0.175±0.007 ^{a}	0.179±0.008 ^{d}	0.256±0.013 ^{d}
AFI2	0.159±0.007 ^{b}	0.219±0.010 ^{b}	0.313±0.014 ^{b}
Ni	0.149±0.007 ^{c}	0.207±0.007 ^{c}	0.295±0.014 ^{c}
Ni+AFI1	0.055±0.006 ^{d}	0.166±0.007 ^{e}	0.238±0.014 ^{e}
Ni+AFI2	0.053±0.006 ^{d}	0.169±0.007 ^{de}	0.244±0.015 ^{de}
	SOD in roots of cv Leningradskaya 6 T		
Control	0.133±0.007 ^{d}	0.136±0.007 ^{d}	0.137±0.005 ^{c}
AFI1	0.150±0.007 ^{ab}	0.156±0.006 ^{bc}	0.157±0.007 ^{ab}
AFI2	0.154±0.007 ^{ab}	0.160±0.007 ^{ab}	0.164 ±0.007 ^{ab}
Ni	0.161±0.006 ^{a}	0.169±0.008 ^{a}	0.173±0.006 ^{a}
Ni+AFI1	0.138±0.008 ^{cd}	0.145±0.008 ^{cd}	0.149±0.005 ^{bc}
Ni+AFI2	0.146±0.006 ^{bc}	0.156±0.006 ^{bc}	0.159±0.011 ^{ab}
	SOD in shoots of cv Leningradskaya 6 T		
Control	0.048±0.004 ^{b}	0.100±0.010 ^{a}	0.140±0.005 ^{a}
AFI1	0.064±0.004 ^{a}	0.095±0.006 ^{a}	0.135±0.005 ^{a}
AFI2	0.071±0.003 ^{a}	0.102±0.007 ^{a}	0.146±0.006 ^{a}
Ni	0.055±0.007 ^{b}	0.083±0.009 ^{b}	0.115±0.004 ^{b}
Ni+AFI1	0.053±0.005 ^{b}	0.068±0.005 ^{c}	0.097±0.006 ^{c}
Ni+AFI2	0.049±0.007 ^{b}	0.072±0.005 ^{c}	0.102±0.005 ^{b}

Control: non-inoculated wheat plants. AFI1: wheat plants inoculated with *B. megaterium* AFI1 (in concentration 5-8×10^{-5} cells mL-1). AFI2: wheat plants inoculated with *P. nicotianae* AFI2 (in concentration 5-8×10^{-5} cells mL^{-1}). Ni: wheat plants grown with Ni concentration 100 μM·L^{-1}. Ni + AFI1: wheat plants with Ni concentration 100 μM·L^{-1} and inoculated with B. megaterium AFI1. Ni + AFI2: wheat plants grown with Ni concentration 100 μM·L-1 and inoculated with P. nicotianae AFI2.

Plant samples were analyzed after 3, 6, 10 days of Ni exposure. Each variant was carried out in three biological replicates with 50 seedlings per replicate after 3 days and with 30 seedlings per replicate after 6, 10 days. Each experiment was repeated 3 times. Bars show ± SEM and different letters show a significant difference among treatments at $p<0.05$ level as determined by Duncan's multiple range test. SOD activity was expressed U mg^{-1}protein min^{-1}

Table S4. Effect of PGPB on POX (U s^{-1} g^{-1} FW) activity of wheat plants under Ni stress.

Experimental variants	Ni exposure time		
	3 days	6 days	10 days
POX in roots of cv Chinese spring S			
Control	55.4±3.3 ^{b}	56.6±3.4 ^{b}	61.9±2.8 ^{c}
AFI1	58.6±3.0 ^{b}	61.3±2.8 ^{b}	70.6±2.1 ^{b}
AFI2	62.5±2.4 ^{b}	61.2±2.7 ^{b}	75.0±3.4 ^{b}
Ni	100.8±4.5 ^{a}	102.7±4.7 ^{a}	116.8±4.9 ^{a}
Ni+AFI1	104.3±3.7 ^{a}	107.9±6.9 ^{a}	118.8±8.2 ^{a}
Ni+AFI2	105.6±4.5 ^{a}	106.9±6.2 ^{a}	119.2±3.7 ^{a}
POX in shoots of cv Chinese spring S			
Control	3.6±0.2 ^{c}	26.8±0.8 ^{d}	32.5±1.3 ^{d}
AFI1	3.7±0.3 ^{c}	29.5±0.6 ^{c}	42.1±1.8 ^{c}
AFI2	3.8±0.2 ^{c}	31.2±1.0 ^{c}	42.3±1.6 ^{c}
Ni	7.8±0.3 ^{b}	55.2±1.1 ^{b}	74.0±3.2 ^{b}
Ni+AFI1	8.1±0.3 ^{ab}	60.6±1.6 ^{a}	82.3±4.3 ^{a}
Ni+AFI2	8.5±0.4 ^{a}	61.3±1.3 ^{a}	83.8±4.3 ^{a}
POX in roots of cv Leningradskaya 6 T			
Control	55.8±2.9 ^{b}	57.7±3.0 ^{b}	65.9±3.7 ^{c}
AFI1	61.1±2.7 ^{b}	62.4±3.0 ^{b}	72.2±4.8 ^{c}
AFI2	61.5±3.1 ^{b}	62.4±3.4 ^{b}	72.4 ±3.4 ^{c}
Ni	96.4±6.1 ^{a}	99.0±4.9 ^{a}	101.7±5.5 ^{b}
Ni+AFI1	100.0±8.4 ^{a}	103.1±5.3 ^{a}	109.3±5.5 ^{a}
Ni+AFI2	97.6±7.7 ^{a}	101.0±3.9 ^{a}	105.7±4.1 ^{ab}
POX in shoots of cv Leningradskaya 6 T			
Control	2.1±0.2 ^{c}	30.3±1.8 ^{c}	36.1±2.3 ^{d}
AFI1	2.2±0.2 ^{c}	32.0±1.5 ^{c}	38.4±2.1 ^{d}
AFI2	2.3±0.2 ^{c}	32.0±1.9 ^{c}	37.6±2.1 ^{d}
Ni	6.3±0.5 ^{a}	47.3±1.7 ^{b}	55.3±2.4 ^{c}
Ni+AFI1	5.9±0.3 ^{ab}	51.2±2.7 ^{a}	64.3±2.2 ^{b}
Ni+AFI2	5.8±0.4 ^{b}	53.4±2.0 ^{a}	68.9±3.2 ^{a}

Control: noninoculated wheat plants grown in Knop medium. AFI1: wheat plants grown in Knop solution inoculated with *Bacillus megaterium* AFI1, in concentration 5-8×10^{-5} cells mL^{-1}. AFI2: wheat plants grown in Knop solution inoculated with *Paenibacillus nicotianae* AFI2 in concentration 5-8×10^{-5} cells mL^{-1}. Ni: wheat plants grown in Knop solution with Ni concentration 100 μM L^{-1}. Ni + AFI1: wheat plants grown in Knop solution with Ni concentration 100 μM·L^{-1} and inoculated with *B. megaterium* AFI1 in concentration 5-8×10^{-5} cells mL^{-1}. Ni + AFI2: wheat plants grown in Knop solution with Ni concentration 100 μM·L^{-1} and inoculated with *P. nicotianae* AFI2 in concentration 5-8×10^{-5} cells mL^{-1}.

Plant samples were analyzed after 3, 6, 10 days of Ni exposure. Each variant was carried out in three biological replicates with 50 seedlings per replicate after 3 days and with 30 seedlings per replicate after 6, 10 days. Each experiment was repeated 3 times. FW - fresh weight of shoots and roots. Bars show ± SEM and different letters show a significant difference among treatments at $p<0.05$ level as determined by Duncan's multiple range test. POX activity was expressed in units, POX activity was expressed in units, each representing the absorbance value per $second^{-1}$ per g^{-1} FW.

Table S5. Effect of PGPB on APX activity (nM ascorbate mg^{-1} protein min^{-1}) of wheat plants under Ni stress.

Experimental variants	Ni exposure time		
	3 days	6 days	10 days
	APX in roots of cv Chinese spring S		
Control	5.43±0.31 ^{e}	5.07±0.12 ^{e}	5.60±0.37 ^{d}
AFI1	6.23±0.38 ^{d}	6.61±0.09 ^{c}	6.51±0.23 ^{c}
AFI2	6.43±0.36 ^{d}	6.68±0.09 ^{c}	6.79±0.44 ^{c}
Ni	9.40±0.48 ^{a}	9.22±0.09 ^{a}	9.05±0.32 ^{a}
Ni+AFI1	7.37±0.41 ^{b}	7.47±0.11 ^{b}	7.75±0.37 ^{b}
Ni+AFI2	6.93±0.38 ^{c}	6.42±0.06 ^{d}	6.58±0.28 ^{c}
	APX in shoots of cv Chinese spring S		
Control	2.29±0.10 ^{d}	2.15±0.07 ^{d}	2.09±0.07 ^{d}
AFI1	2.32±0.06 ^{d}	2.35±0.06 ^{c}	2.41±0.13 ^{bc}
AFI2	2.26±0.06 ^{d}	2.28±0.07 ^{c}	2.30±0.15 ^{bc}
Ni	3.41±0.08 ^{a}	4,75±0.10 ^{a}	5.24±0.17 ^{a}
Ni+AFI1	3,28±0.06 ^{b}	2,84±0.07 ^{b}	2.48±0.09 ^{b}
Ni+AFI2	3,12±0.06 ^{c}	2,78±0.06 ^{b}	2.27±0.07 ^{c}
	APX in roots of cv Leningradskaya 6 T		
Control	4.03±0.10 ^{d}	4.14±0.06 ^{f}	4.30±0.24 ^{d}
AFI1	5.21±0.08 ^{c}	5.12±0.06 ^{d}	4.95±0.19 ^{c}
AFI2	5.10±0.09 ^{c}	4.96±0.11 ^{e}	4.76±0.25 ^{c}
Ni	7.39±0.06 ^{a}	7.26±0.06 ^{a}	7.16±0.40 ^{a}
Ni+AFI1	6.65±0.08 ^{b}	6.51±0.10 ^{b}	6.50±0.30 ^{b}
Ni+AFI2	6.38±0.07 ^{b}	6.22±0.07 ^{c}	6.18±0.29 ^{b}
	APX in shoots of cv Leningradskaya 6 T		
Control	1.24±0.05 ^{e}	1.37±0.05 ^{d}	1.34±0.06 ^{e}
AFI1	1.41±0.05 ^{cd}	1.45±0.07 ^{d}	1.54±0.09 ^{d}
AFI2	1.64±0.08 ^{b}	1.72±0.09 ^{c}	1.86±0.09 ^{c}
Ni	4.82±0.07 ^{a}	4.27±0.07 ^{a}	3.75±0.18 ^{a}
Ni+AFI1	1.50±0.06 ^{c}	1.83±0.08 ^{b}	2.39±0.13 ^{b}
Ni+AFI2	1.36±0.08 ^{d}	1.72±0.06 ^{c}	2.33±0.16 ^{b}

Control: noninoculated wheat plants grown in Knop medium. AFI1: wheat plants grown in Knop solution inoculated with *Bacillus megaterium* AFI1, in concentration 5-8×10^{-5} cells mL^{-1}. AFI2: wheat plants grown in Knop solution inoculated with *Paenibacillus nicotianae* AFI2 in concentration 5-8×10^{-5} cells mL^{-1}. Ni: wheat plants grown in Knop solution with Ni concentration 100 μM L^{-1}. Ni + AFI1: wheat plants grown in Knop solution with Ni concentration 100 μM·L^{-1} and inoculated with *B. megaterium* AFI1 in concentration 5-8×10^{-5} cells mL^{-1}. Ni + AFI2: wheat plants grown in Knop solution with Ni concentration 100 μM·L^{-1} and inoculated with *P. nicotianae* AFI2 in concentration 5-8×10^{-5} cells mL^{-1}.

Plant samples were analyzed after 3, 6, 10 days of Ni exposure. Each variant was carried out in three biological replicates with 50 seedlings per replicate after 3 days and with 30 seedlings per replicate after 6, 10 days. Each experiment was repeated 3 times. Bars show ± SEM and different letters show a significant difference among treatments at $p<0.05$ level as determined by Duncan's multiple range test. APX activity was expressed in nM ascorbate oxidized per minute per mg protein.

Table S6. Effect of PGPB on lipid oxidation status LPO (measured in MDA, μM g^{-1}FW) of wheat plants under Ni stress.

Experimental variants	Ni exposure time		
	3 days	6 days	10 days
	LPO in roots of cv Chinese spring S		
Control	2.54±0.16 c	2.34±0.12 c	1.88±0.12 c
AFI1	2.48±0.13 c	2.40±0.11 c	1.83±0.13 c
AFI2	2.54±0.09 c	2.32±0.09 c	1.86±0.12 c
Ni	3.86±0.22 a	3.52±0.10 a	3.36±0.25 a
Ni+AFI1	3.54±0.13 b	2.43±0.11 c	1.96±0.10 c
Ni+AFI2	3.70±0.08 ab	2.79±0.16 b	2.53±0.16 b
	LPO in shoots of cv Chinese spring S		
Control	3.74±0.16 c	3.52±0.16 c	3.42±0.19b
AFI1	2.48±0.13 e	2.35±0.15 e	2.19±0.16d
AFI2	2.84±0.14 d	2.63±0.18 d	2.50±0.16 c
Ni	5.63±0.20 a	5.33±0.25 a	4.33±0.27 a
Ni+AFI1	4.22±0.15 b	3.85±0.17 b	3.40±0.20 b
Ni+AFI2	4.38±0.21 b	3.98±0.20 b	3.57±0.16 b
	LPO in roots of cv Leningradskaya 6 T		
Control	2.06±0.16 c	2.11±0.12 b	1.56±0.09 c
AFI1	1.96±0.11 c	2.09±0.15 b	1.52±0.10 c
AFI2	2.04±0.17 c	1.96±0.10 b	1.58±0.09 c
Ni	3.11±0.18 a	2.75±0.17 a	2.54±0.13 a
Ni+AFI1	2.69±0.10 b	2.06±0.13 b	1.78±0.11 b
Ni+AFI2	2.75±0.16 b	2.13±0.15 b	1.96±0.13 b
	LPO in shoots of cv Leningradskaya 6 T		
Control	3.04±0.12 c	2.72±0.11 c	2.53±0.12 bc
AFI1	2.71±0.12 d	2.43±0.09 d	2.43±0.15 c
AFI2	2.77±0.10 d	2.54±0.12 cd	2.55±0.15 bc
Ni	4.16±0.18 a	3.86±0.18 a	3.22±0.15 a
Ni+AFI1	3.47±0.27 b	3.08±0.14 b	2.63±0.12 bc
Ni+AFI2	3.52±0.18 b	3.21±0.14 b	2.71±0.18 b

Control: noninoculated wheat plants grown in Knop medium. AFI1: wheat plants grown in Knop solution inoculated with *Bacillus. megaterium* AFI1, in concentration 5-8×10^{-5} cells mL^{-1}. AFI2: wheat plants grown in Knop solution inoculated with *Paenibacillus nicotianae* AFI2 in concentration 5-8×10^{-5} cells mL^{-1}. Ni: wheat plants grown in Knop solution with Ni concentration 100 μM L^{-1}. Ni + AFI1: wheat plants grown in Knop solution with Ni concentration 100 μM·L^{-1} and inoculated with *B. megaterium* AFI1 in concentration 5-8×10^{-5} cells mL^{-1}. Ni + AFI2: wheat plants grown in Knop solution with Ni concentration 100 μM·L^{-1} and inoculated with *P. nicotianae* AFI2 in concentration 5-8×10^{-5} cells mL^{-1}.

Plant samples were analyzed after 3, 6, 10 days of Ni exposure. Each variant was carried out in three biological replicates with 50 seedlings per replicate after 3 days and with 30 seedlings per replicate after 6, 10 days. Each experiment was repeated 3 times. FW - fresh weight of shoots and roots. Bars show ± SEM and different letters show a significant difference among treatments at $p<0.05$ level as determined by Duncan's multiple range test. The concentration of TBA-reactive products was expressed in MDA μM g^{-1} FW.

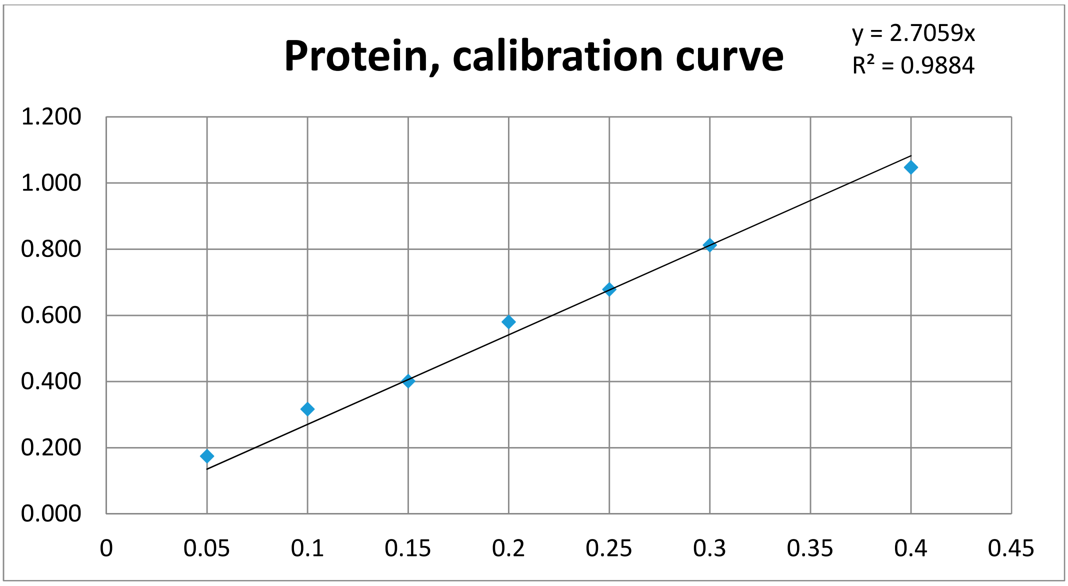

Figure S3. Protein calibration curve.

Total soluble protein was determined after [69], with bovine serum albumin (BSA) is being used as the standard.

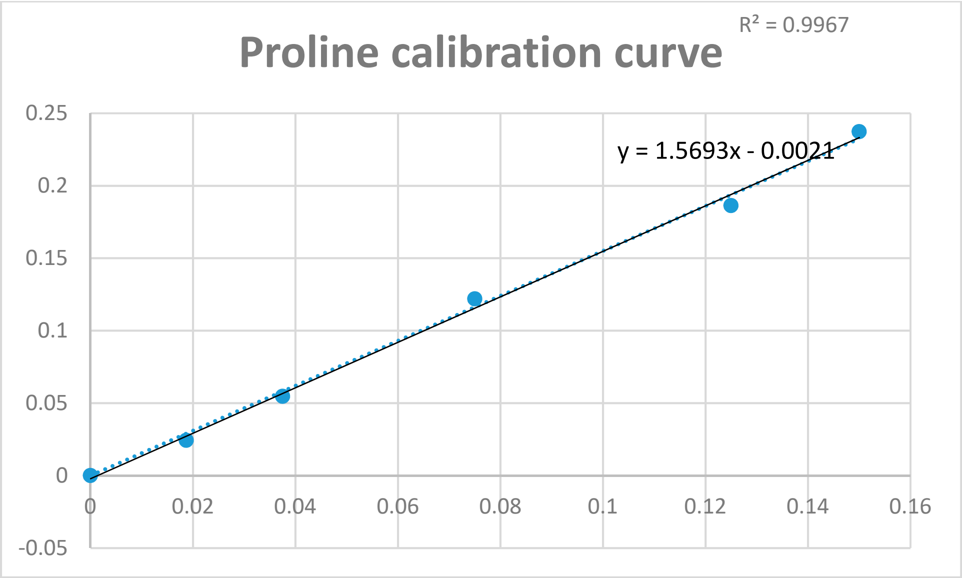

Figure S4. Proline calibration curve

The content of proline was determined following by Bates [75].
